# Targeting the up-regulated CNOT3 reverses therapeutic resistance and metastatic progression of EGFR-mutant non-small cell lung cancer

**DOI:** 10.1038/s41420-023-01701-w

**Published:** 2023-11-02

**Authors:** Lin Jing, Meng-En Zhai, Mei-Rui Qian, Yi-Ming Li, Ming-Wei Han, Kun Wang, Wan Huang, Gang Nan, Jian-Li Jiang

**Affiliations:** 1 Department of Cell Biology, National Translational Science Center for Molecular Medicine, Fourth Military Medical University, Xi’an, 710032 Shaanxi China; 2 Department of Cardiovascular Surgery, Xijing Hospital, Air Force Medical University, Xi’an, 710032 Shaanxi China

**Keywords:** Non-small-cell lung cancer, Cancer therapeutic resistance, Metastasis

## Abstract

Lung cancer is the leading cause of cancer-related mortality worldwide. CNOT3, a subunit of the CCR4-NOT complex, has recently been suggested to be overexpressed in lung cancer and involved in tumor malignancy. However, its precise role and the underlying mechanisms still need to be fully revealed. In the present study, we found in lung cancer cells the expression of CNOT3 could be regulated by EGFR signaling pathway and c-Jun, a transcription factor downstream of EGFR, transcriptionally regulated its expression. Interestingly, CNOT3 could inversely regulate the expression of c-Jun via modulating its translation. Thus, a feedback loop existed between c-Jun and CNOT3. CNOT3 reduction post EGFR blockade facilitated the drug-induced cell death, and simultaneously inhibited cell proliferation via impacting TSC1/mTOR axis. Whereas, further up-regulation of the CNOT3 expression was observed in gefitinib-resistant cells, which dampened gefitinib sensitivity. Mechanically, the elevation of CNOT3 was induced by the bypass activation of HER2/c-Jun signaling. Depleting CNOT3 in vitro and in vivo sensitized the drug-resistant cells to gefitinib treatment and inhibited metastatic progression. These results give novel insights into the role of CNOT3 in lung cancer malignancy and provide a theoretical basis for the development of therapeutic strategies to solve acquired resistance to EGFR-TKIs.

## Introduction

Lung cancer remains to be the leading cause of cancer-related mortality worldwide and the major histological subtype is non-small cell lung cancer (NSCLC) [1, 2]. It has been illustrated that almost two-thirds of patients with NSCLC harbor oncogenic driver mutations, among which somatic activating mutations in epidermal growth factor receptor (EGFR) are frequently detected [3]. The overactivation of EGFR contributes to cancer malignancy via promoting proliferation, migration as well as survival of the tumor cells [4]. EGFR tyrosine kinase inhibitors (EGFR-TKIs) which are permitted to treat NSCLC patients harboring activating *EGFR* mutations can block EGFR and lead to great improvement in survival [5]. However, acquired resistance eventually occurs and patients ultimately suffer disease progression [6]. Several acquired resistance mechanisms have been elucidated such as second-site mutations in *EGFR*, oncogene amplification or loss, activation of EGFR downstream pathway or bypass activation [7–10]. Whereas, these mechanisms tend more to illustrate the in-situ reoccurrence of the tumor. Metastatic progression results from the surviving tumor cells escaping from the damaged site. Drug resistance and metastatic progression are suggested to determine the outcome of a patient with cancer jointly. Some evidences have shown that signaling pathway that steer resistance to therapeutic treatment can intersect with the signaling of cell invasion, but the shared mechanisms still need to be fully discovered including in EGFR-TKIs resistance-induced tumor progression [11].

CNOT3 is a subunit of carbon catabolite repression 4 (CCR4)-negative on TATA-less (NOT), a multimeric complex which is conserved in all eukaryotes and responsible for RNA metabolism [12]. The molecular functions of this subunit have been addressed in yeast that it is important for mRNA translatability and polyribosome levels [13]. In mammalian, CNOT3 is also demonstrated to promote mRNA decay [14]. Currently, the studies on CNOT3 mainly focus on how it is involved in physiological activities or diseases. Its participation in energy metabolism [15, 16], cell development [17] and cell death [18] has already been demonstrated. CNOT3 is also found to be aberrantly expressed in tumor and to play a role in promoting tumor formation and progression [19, 20]. Additionally, it is reported that CNOT3 contributes to cisplatin resistance in both NSCLC and renal cancer [21, 22]. Even so, the function of CNOT3 in cancer is still not fully known and its involvement in the resistance to other anti-tumor therapeutics remains elusive.

In this study, we showed that the expression of CNOT3 could be regulated by EGFR signaling in lung cancer. Besides, a feedback loop existed between c-Jun and CNOT3. CNOT3 was involved to maintain cell proliferation and survival in EGFR signaling. Thus, further up-regulation of CNOT3, which was due in part to HER2 overexpression-induced dysregulation of the c-Jun/CNOT3 axis, dampened gefitinib sensitivity in lung cancer cells. Depleting CNOT3 could enhance the anti-tumor effect of gefitinib and inhibit tumor metastasis, suggesting CNOT3 is a significant factor to steer both gefitinib resistance and metastatic progression. Together, our findings will give novel insights into the role of CNOT3 in lung cancer malignancy and provide a promising strategy to resolve both EGFR-TKIs resistance and tumor progression.

## Results

### EGFR signaling regulates CNOT3 expression

**Fig. 1:**
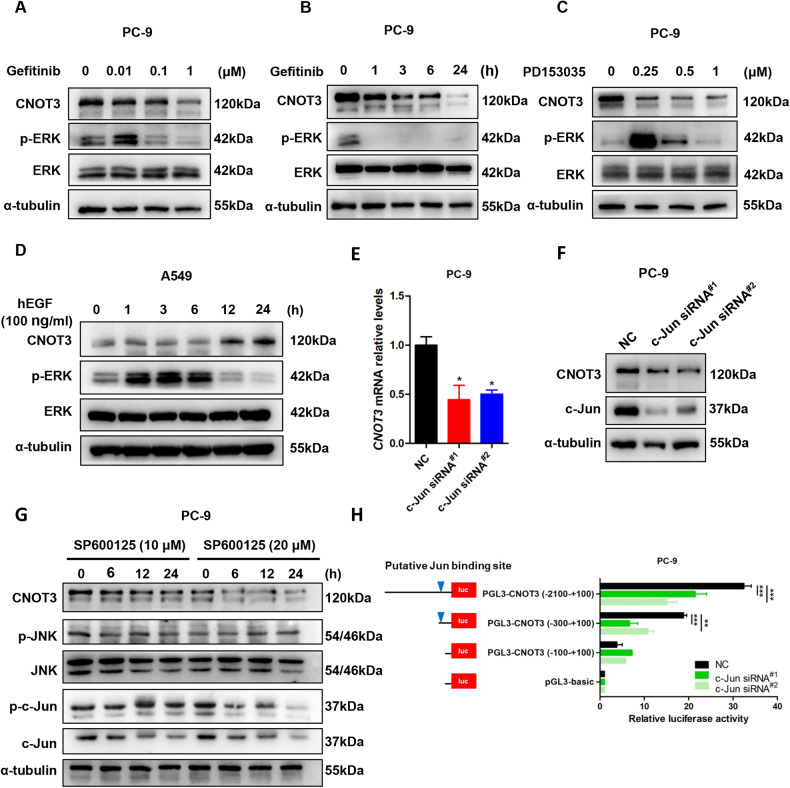
EGFR signaling regulates CNOT3 expression. **A** PC-9 cells were treated with gefitinib for 24 h, then cells were subjected to western blotting to assess protein expression levels. **B** PC-9 cells were treated with gefitinib for the indicated time, then cells were subjected to western blotting to assess protein expression levels. **C** PC-9 cells were treated with PD153035 for 24 h, then cells were subjected to western blotting to assess protein expression levels. **D** A549 cells were treated with human EGF for the indicated time after serum starvation for 12 h, then cells were subjected to western blotting to assess protein expression levels. **E,** **F** PC-9 cells were transfected with a control or c-Jun siRNA. Then *CNOT3* mRNA levels in PC-9 cells were measured by q-PCR (E). Cells were subjected to western blotting to assess protein expression levels (F). **G** PC-9 cells were treated with SP600125 for the indicated time, then cells were subjected to western blotting to assess protein expression levels. **H** PC-9 cells were transfected with c-Jun siRNA and luciferase reporter plasmids containing truncated *CNOT3* promoter, followed by luciferase reporter assays. Data are shown as the mean±S.E.M. *n* = 3. **E** One-way ANOVA with Tukey post hoc test. **H** Two-way ANOVA and Bonferroni post hoc test. ^***^P < 0.001, ^**^P < 0.01 or ^*^P < 0.05 for comparisons between the indicated groups.

Alterations in CNOT3 expression can be observed in many types of cancer which subsequently induces alterations in cell functions and contributes to tumor malignancy [19]. However, how CNOT3 expression is regulated is still unclear. We consulted the RNA-seq datasets of the TCGA database and noticed CNOT3 expression was positively correlated with the expression of some ErbB family of receptor tyrosine kinases such as EGFR, ErbB-2 (neu, HER2) and ErbB-3 (HER3) in lung adenocarcinoma (Supplementary Fig. 1A, B, C). We then employed inhibitors to block EGFR and found the expression of CNOT3 was down-regulated by EGFR inhibitors (gefitinib and PD135035) in PC-9 and HCC827 cells, which indicated the expression of CNOT3 might be regulated by EGFR signaling (Fig. 1A–C and Supplementary Fig. 1D). To confirm this, we used EGF to further stimulate EGFR in A549 cells, and noticed the expression of CNOT3 was up-regulated at both protein and mRNA level, especially at 24 h post EGF treatment (Fig. 1D and Supplementary Fig. 1E). We also employed a HER2 inhibitor tucatinib to treat PC-9 cells, but the results showed that it did not affect CNOT3 expression (Supplementary Fig. 1F). Collectively, the above data indicated that the expression of CNOT3 could be regulated by EGFR signaling.

EGFR activation initiates signaling transduction and activates transcription factors such as c-Fos, c-Jun and c-myc to regulate gene expression [23]. To further explore how CNOT3 expression is regulated, we used JASPAR (http://jaspar.genereg.net) and performed sequence analysis. The results showed c-Fos and c-Jun were potential transcription factors. To further validate, the expression of c-Fos was first depleted, but no changes in CNOT3 expression were noticed (data not shown). Then, we depleted the expression of c-Jun, and found that CNOT3 expression significantly decreased at both mRNA and protein levels in PC-9 and HCC827 cells (Fig. 1E, F and Supplementary Fig. 2A). Given that c-Jun can be phosphorylated and then activated by c-Jun amino-terminal kinases (JNKs), a group of mitogen-activated protein kinases (MAPKs) working downstream of EGFR [24], we next used JNK inhibitor SP600125 to inhibit c-Jun phosphorylation and examined the changes in CNOT3 expression. The results showed that CNOT3 expression decreased in a dose- and time-dependent manner following SP600125 treatment (Fig. 1G and Supplementary Fig. 2B). The decreases in the expression of phosphorylated JNK and phosphorylated c-Jun following gefitinib treatment were also observed, and the trends were consistent with that of CNOT3 (Supplementary Fig. 2C). Given that the predicted c-Jun binding site was at −192 bp to −179 bp upstream of the *CNOT3* transcription start site (TSS) (Supplementary Fig. 2D), we next constructed and transfected cells with a series of pGL3 reporter plasmids which contained sequential deletion of the 5′-flanking region upstream of *CNOT3*, and knocked down the expression of c-Jun at the same time. The results showed that c-Jun depletion led to decreases in luciferase activities in both PC-9 and HCC829 cells, while sequence deletions from −2100 bp to −100 bp upstream of the *CNOT3* TSS abrogated the reduction in luciferase activity induced by c-Jun depletion (Fig. 1H and Supplementary Fig. 2E). In summary, these data indicated that CNOT3 could be transcriptionally regulated by c-Jun, a downstream transcription factor in EGFR signaling pathway.

### A feedback loop exists between c-Jun and CNOT3

**Fig. 2:**
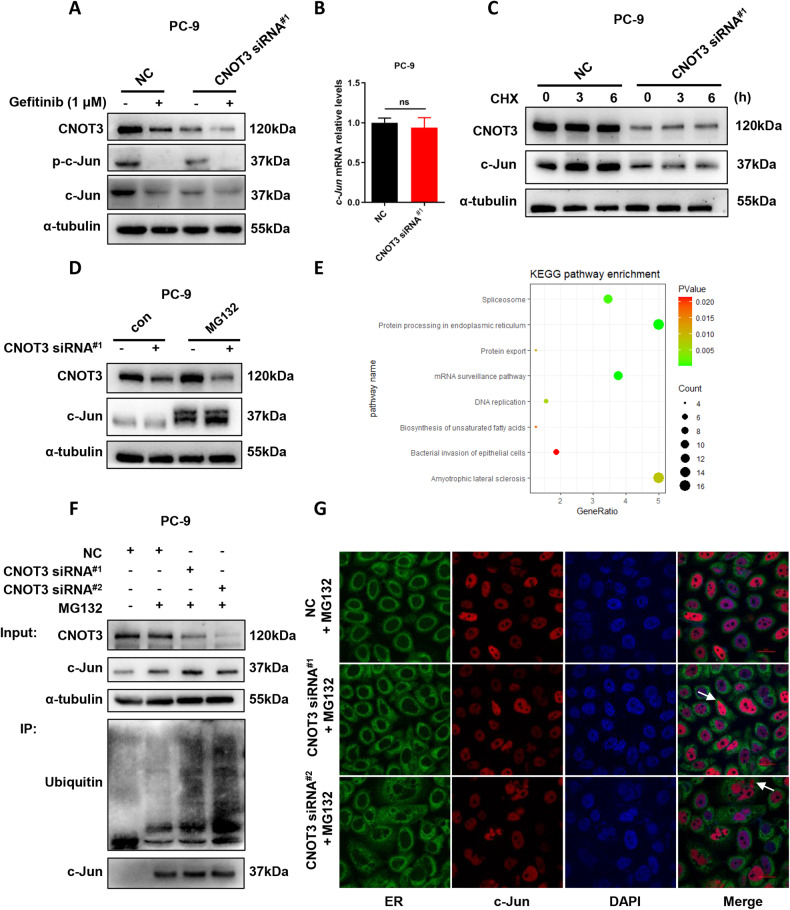
A feedback loop exists between c-Jun and CNOT3. **A,** **B** PC-9 cells were transfected with a control or CNOT3 siRNA^#1^. Twenty-four hours after transfection, cells were treated with gefitinib for 24 h. Then cells were subjected to western blotting to assess protein expression levels (**A**). *c-Jun* mRNA levels in PC-9 cells were measured by q-PCR (**B**). **C,** **D** PC-9 cells were transfected with a control or CNOT3 siRNA^#1^. Twenty-four hours after transfection, cells were treated with CHX (10 μM) for the indicated time, then cells were subjected to western blotting to assess protein expression levels (**C**). Twenty-four hours after transfection, cells were treated with MG132 (1 μM) for 9 h, then cells were subjected to western blotting to assess protein expression levels (**D**). **E** Genes that were up-regulated over 1.5-fold in the CNOT3-depleted PC-9 cells were identified using KEGG analysis. **F,** **G** PC-9 cells were transfected with a control or CNOT3 siRNA. Twenty-four hours after transfection, cells were treated with MG132 for 9 h. Cell lysates were immunoprecipitated with an anti-c-Jun antibody and analyzed by western blotting with the indicated antibodies (**F**). Representative images of PC-9 cells stained with anti-c-Jun antibody, ER tracker and DAPI (**G**). Data are shown as the mean±S.E.M. *n* = 3. **B** Student’s *t*-test.

It is conceivable that gefitinib treatment will inhibit c-Jun phosphorylation. However, we noticed that total amount of the c-Jun protein also decreased after gefitinib treatment (Fig. 2A), which is consistent with a previous study [25]. Interestingly, we found that c-Jun expression reduced when CNOT3 was knocked down (Fig. 2A and Supplementary Fig. 3A). Thus, we speculated CNOT3 was able to regulate the expression of c-Jun. Considering that CNOT3 is notable for post-transcriptionally regulating gene expressions via influencing mRNA degradation, we first detected whether CNOT3 depletion would impact c-Jun expression at mRNA level. To our surprise, no obvious changes were found in *c-Jun* mRNA expression (Fig. 2B).

To elucidate how c-Jun expression is regulated by CNOT3, we performed proteomic analysis and made comparisons between the CNOT3-depleted cells and the wild-type cells. Kyoto Encyclopedia of Genes and Genomes (KEGG) pathway enrichment analysis showed that in CNOT3-depleted cells, the expression of genes related to protein processing in endoplasmic reticulum (ER) and proteasome were up-regulated (Fig. 2E and Supplementary Fig. 3D). We employed cycloheximide (CHX) to inhibit protein synthesis, and found that the c-Jun protein degraded faster when CNOT3 was knocked down (Fig. 2C and Supplementary Fig. 3B). More ubiquitin-conjugated c-Jun was detected in cells with CNOT3 depletion when MG132 treatment was used to inhibit proteasome-mediated protein degradation (Fig. 2F). Correspondingly, more c-Jun was detected in CNOT3-depleted cells when protein degradation was inhibited (Fig. 2D and Supplementary Fig. 3C). We also performed immunocytochemistry to observe the location of c-Jun in CNOT3-depleted cells. The results showed that in CNOT3-depleted cells, c-Jun protein aggregated in the ER especially when cells were treated with MG132 (Fig. 2G and Supplementary Fig. 3E). Thus, the data aforementioned indicated that CNOT3 depletion would cause c-Jun aggregation, which would trigger ER-associated degradation (ERAD) to erase the aggregated protein and ultimately induced a reduction in c-Jun expression in cells.

In short, the above findings suggested there existed a feedback loop between c-Jun and CNOT3.

### CNOT3 helps to maintain cell proliferation and survival in EGFR signaling

**Fig. 3:**
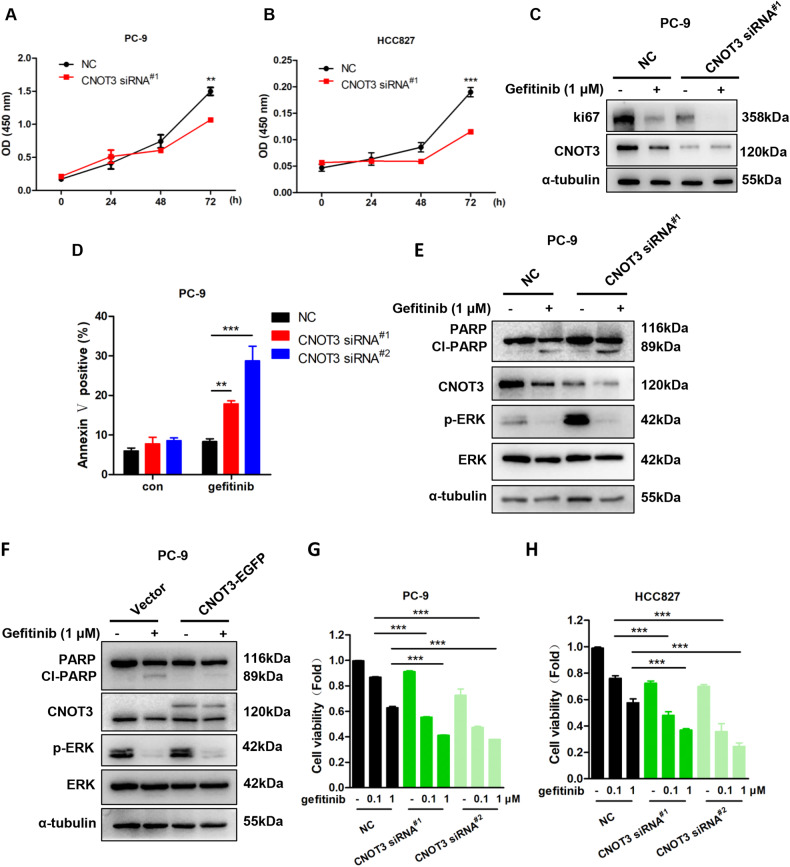
CNOT3 helps to maintain cell proliferation and survival in EGFR signaling. **A,** **B** PC-9 and HCC827 cells were transfected with a control or CNOT3 siRNA^#1^ and cell proliferation was measured via the CCK-8 assay. **C–E** PC-9 cells were transfected with a control or CNOT3 siRNA. Twenty-four hours after transfection, cells were treated with gefitinib for 24 h. Cells were subjected to western blotting to assess protein expression levels (**C**, **E**). Cell death was analyzed via the flow cytometry (**D**). **F** PC-9 cells stably transfected with the empty vector or the recombinant CNOT3 plasmid were treated with gefitinib for 24 h, then cells were subjected to western blotting to assess protein expression levels. **G,** **H** PC-9 or HCC827 cells were transfected with a control or CNOT3 siRNA. Twenty-four hours after transfection, cells were treated with gefitinib for 24 h. PC-9 cell viability was measured via the CCK-8 assay (**G**). HCC827 cell viability was measured via the CCK-8 assay (**H**). Data are shown as the mean±S.E.M. *n* = 3. **A,** **B** and **D** Two-way ANOVA and Bonferroni post hoc test. **G,** **H** One-way ANOVA with Tukey post hoc test. ^***^P < 0.001 or ^**^P < 0.01 for comparisons between the indicated groups.

CNOT3 is suggested to be important in maintaining cell proliferation and survival [18, 19]. We validated in PC-9 and HCC827 cells that CNOT3 depletion hindered cell proliferation (Fig. 3A, B). Considering a reduction in CNOT3 expression after EGFR blockade, we confirmed when the expression of CNOT3 was further reduced, the gefitinib-induced cell proliferation inhibition could be enhanced (Fig. 3C). In addition, the apoptotic cell death was accelerated in CNOT3-depleted cells post gefitinib treatment (Fig. 3D, E, Supplementary Fig. 4A, B). When CNOT3 was overexpressed, the gefitinib-induced apoptosis could be alleviated (Fig. 3F). The CCK-8 assay was also performed and verified that CNOT3 knocking down further enhanced the inhibitory efficacy of gefitinib (Fig. 3G, H). Based on this, we concluded that CNOT3 was involved to maintain cell proliferation and survival in EGFR signaling.

**Fig. 4:**
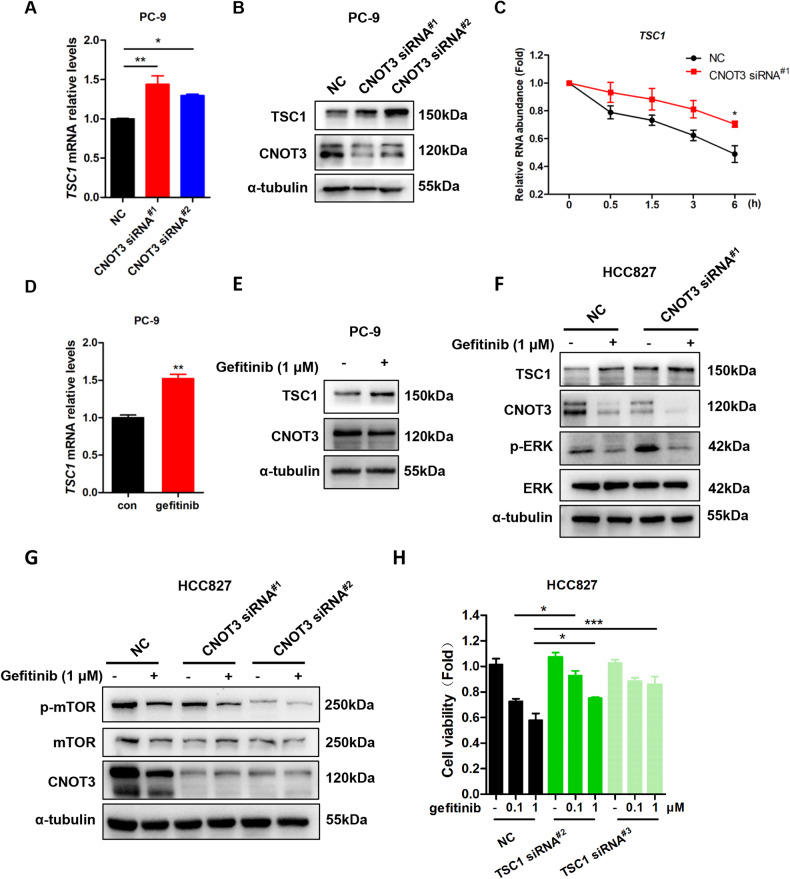
CNOT3 regulates cell proliferation via influencing TSC1/mTOR axis. **A,** **B** PC-9 cells were transfected with a control or CNOT3 siRNA. *TSC1* mRNA levels in PC-9 cells were measured by q-PCR (**A**). Cells were subjected to western blotting to assess protein expression levels (**B**). **C** PC-9 cells were transfected with a control or CNOT3 siRNA^#1^ followed by Act. D treatment. Relative mRNA levels were determined by q-PCR at the indicated time after Act. D treatment and normalized to the *GAPDH* mRNA level. mRNA level prior to Act. D treatment (0 h) was set to 1. **D,** **E** PC-9 cells were treated with gefitinib for 24 h, *TSC1* mRNA levels in PC-9 cells were measured by q-PCR. Cells were subjected to western blotting to assess protein expression levels (**E**). **F,** **G** HCC827 cells were transfected with a control or CNOT3 siRNA. Twenty-four hours after transfection, cells were treated with gefitinib for 24 h. Cells were subjected to western blotting to assess protein expression levels. **H** HCC827 cells were transfected with a control or TSC1 siRNA. Twenty-four hours after transfection, cells were treated with gefitinib for 24 h. Then cell viability was measured via the CCK-8 assay. Data are shown as the mean±S.E.M. *n* = 3. **A,** **H** One-way ANOVA with Tukey post hoc test. **C** Two-way ANOVA and Bonferroni post hoc test. **D** Student’s *t*-test. ^***^P < 0.001, ^**^P < 0.01 or ^*^P < 0.05 for comparisons between the indicated groups.

In previous study, some genes that play function both in cell death and cell cycle arrest were shown to be differentially expressed following CNOT3 depletion, and Tuberous sclerosis complex 1 (TSC1) was included [26]. TSC1 is demonstrated to interact with TSC2 to inhibit Rapamycin complex 1 (mTORC1), which is pivotal in controlling cell growth and metabolisms in response to nutrients, growth factors (such as EGF), cellular energy, and stress [27, 28]. We verified when CNOT3 was knocked down, the expression of TSC1 was up-regulated at both mRNA and protein levels (Fig. 4A, B and Supplementary Fig. 4C). We also found that the half-life of *TSC1* mRNA was prolonged in CNOT3-knockdown cells, suggesting the increased TSC1 expression due in part to the inhibition of mRNA decay (Fig. 4C). As CNOT3 expression was down-regulated following gefitinib treatment, we next detected the expression of TSC1 at the same condition and the results showed an increase in its expression (Fig. 4D, E, F and Supplementary Fig. 4C). mTOR phosphorylation was also examined following CNOT3 depletion. Our results showed that knocking down CNOT3 would inhibit mTOR phosphorylation and it was more significant when in combination with gefitinib (Fig. 4G and Supplementary Fig. 4D). To our surprise, we noticed TSC1 depletion could not reduce gefitinib-triggered apoptosis (Supplementary Fig. 4E). However, largely because cell proliferation was promoted in the TSC1-depleted cells, knocking down TSC1 rescued the decrease in cell viability induced by gefitinib (Fig. 4H, Supplementary Fig. 4E, F). Together, the above findings indicated that in response to EGFR blockade, CNOT3 down-regulation could influence TSC1/mTOR axis and this mainly hindered cell proliferation.

### The c-Jun/CNOT3 axis is dysregulated in PC-9 GR cells

**Fig. 5:**
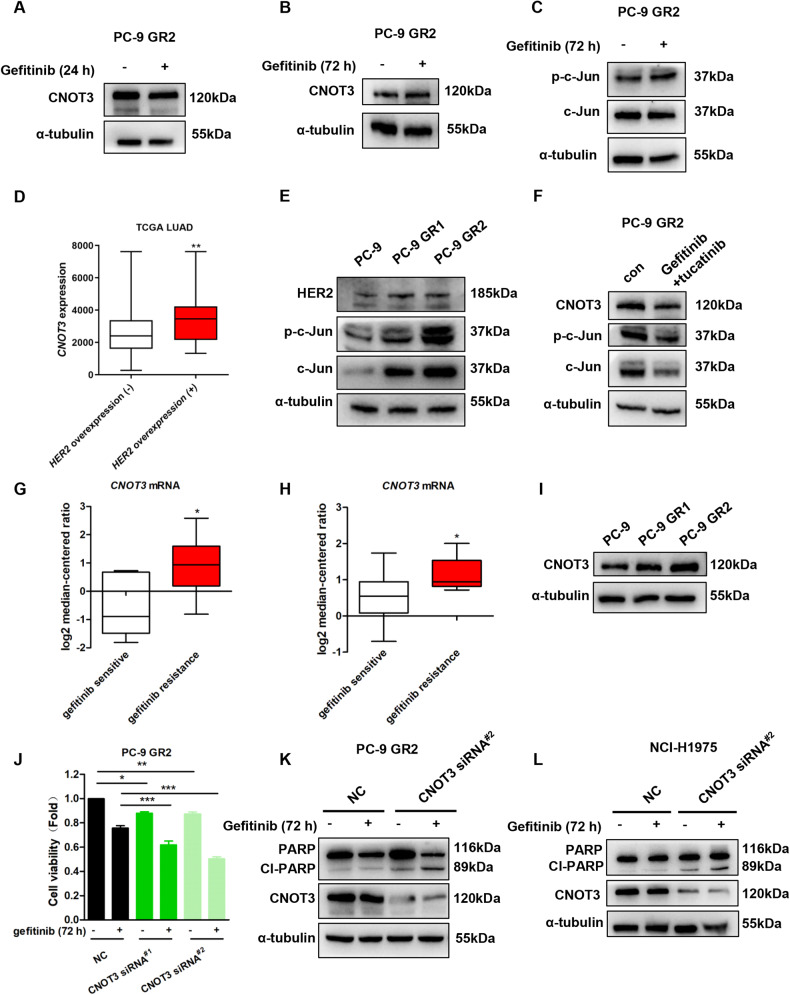
Dysregulation of the c-Jun/CNOT3 axis contributes to gefitinib resistance in NSCLC. **A–C** PC-9 GR2 cells were treated with gefitinib for the indicated time, then cells were subjected to western blotting to assess protein expression levels. **D** Comparison of *CNOT3* mRNA level between lung adenocarcinoma tissues with or without HER2 overexpression downloaded from TCGA database, ***n*** = 32 and 479, respectively. **E** PC-9, PC-9 GR1 and PC-9 GR2 cells were subjected to western blotting to assess protein expression levels. **F** PC-9 GR2 cells were treated with gefitinib (1 μM) and tucatinib (1 μM) for 24 h, then cells were subjected to western blotting to assess protein expression levels. **G,** **H** Comparison of *CNOT3* mRNA level between gefitinib-sensitive cells and gefitinib-resistant cells downloaded from two different datasets. In Zhou cell line, gefitinib sensitive indicates IC_50_ less than 0.4 μM (*n* = 5) and gefitinib resistance indicates IC_50_ more than 4 μM (*n* = 35) (**G**). In Coldren cell line, gefitinib sensitive indicates IC_50_ less than 0.5 μM (*n* = 9) and gefitinib resistance indicates IC_50_ more than 4.5 μM (*n* = 11) (**H**). **I** PC-9, PC-9 GR1 and PC-9 GR2 cells were subjected to western blotting to assess protein expression levels. **J–L** PC-9 GR2 or H1975 cells were transfected with a control or CNOT3 siRNA. Twenty-four hours after transfection, cells were treated with gefitinib for 72 h. Cell viability was measured via the CCK-8 assay in PC-9 GR2 cells (**J**). PC-9 GR2 cells were subjected to western blotting to assess protein expression levels (**K**). H1975 cells were subjected to western blotting to assess protein expression levels (**L**). Data are shown as the mean±S.E.M. *n* = 3. **D,** **G** and **H** Mann–whitney U test. **J** One-way ANOVA with Tukey post hoc test. ^***^P < 0.001, ^**^P < 0.01 or ^*^P < 0.05 for comparisons between the indicated groups.

In our study, two cell lines (PC-9 GR1 and PC-9 GR2 cells) which are less sensitive to gefitinib were generated (Supplementary Fig. 5A, B). It’s interesting that unlike the parent cells, no alterations in the expression of CNOT3 were observed in PC-9 GR cells even if we prolonged gefitinib treatment (Fig. 5A, B and Supplementary Fig. 5C). Additionally, the expression of c-Jun as well as phosphorylated c-Jun did not decrease following the long-time gefitinib treatment (Fig. 5C and Supplementary Fig. 5C). Thus, it seemed that the c-Jun/CNOT3 axis was dysregulated in PC-9 GR cells.

*HER2* amplification is reported to cause gefitinib resistance in NSCLC via inducing bypass activation and JNK is one of the major pathways downstream of HER2 [29, 30]. We noticed that the expression of *CNOT3* was higher in lung adenocarcinoma with *HER2* overexpression (Fig. 5D). In addition, our results showed that the expression of HER2 was up-regulated in PC-9 GR cells at both mRNA and protein levels (Fig. 5E and Supplementary Fig. 5D). Accordingly, the expression of c-Jun and phosphorylated c-Jun increased (Fig. 5E). Thus, it was assumable that dysregulation of the c-Jun/CNOT3 axis in gefitinib-resistant cells was a result of HER2 overexpression. We then used tucatinib to figure out whether blocking HER2 would interrupt c-Jun/CNOT3 signaling in PC-9 GR cells. Surprisingly, the results showed that the expression of CNOT3, c-Jun or phosphorylated c-Jun in GR2 cells was not altered by tucatinib at a concentration of 1 μM, though it was able to inhibit the MEK/ERK pathway (Supplementary Fig. 5E). However, when GR2 cells were treated with both gefitinib and tucatinib, the reduction in CNOT3, c-Jun and phosphorylated c-Jun could be observed (Fig. 5F).

### CNOT3 expression level correlates with gefitinib sensitivity in NSCLC

We next explored whether CNOT3 expression correlates with the response of lung cancer cells to gefitinib via consulting a cancer microarray database called Oncomine, and noticed the expression levels of *CNOT3* mRNA in gefitinib-resistant cell lines were higher than that in gefitinib-sensitive cell lines (Fig. 5G, H). Then three cell lines (HCC827, A549 and NCI-H1975) were chosen to verify the results by performing q-PCR (Supplementary Fig. 5F). We also examined CNOT3 expression in PC-9 GR cells. The results showed that comparing with the parent cells, the expression of CNOT3 was up-regulated in both GR1 and GR2 cells (Fig. 5I). We next investigated in vitro whether knocking down the expression of CNOT3 would sensitize PC-9 GR cells to gefitinib. It revealed that CNOT3 depletion enhanced the anti-tumor effect of gefitinib in PC-9 GR cells as long as we prolonged treatment (Fig. 5J, K and Supplementary Fig. 6). We noticed CNOT3 depletion could even sensitize H1975 cells to gefitinib (Fig. 5L).

Thus, the data above indicated that a higher expression level of CNOT3 could contribute to gefitinib resistance in NSCLC.

### Down-regulating CNOT3 overcomes gefitinib resistance and inhibits metastatic progression in vivo

**Fig. 6:**
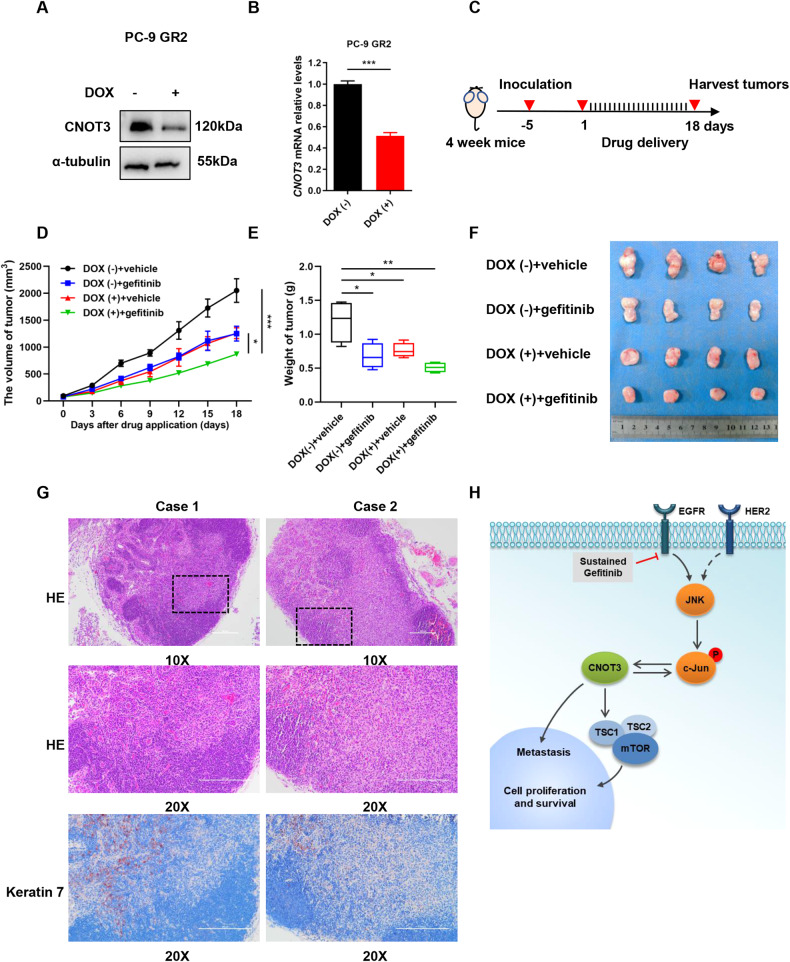
Down-regulating CNOT3 overcomes gefitinib resistance and inhibits metastatic progression in vivo. **A,** **B** PC-9 GR2 cells transfected with the CNOT3-specific shRNA Tet-on inducible plasmid were treated with DOX (2.5 μg/ml) for 48 h. Cells were subjected to western blotting to assess protein expression levels (**A**). *CNOT3* mRNA levels in cells were measured by q-PCR (**B**). **C–G** BALB/c nude mice were inoculated with PC-9 GR2 cells harboring CNOT3-specific shRNA Tet-on inducible plasmid. A schematic view of the treatment plan (**C**). Plots of tumor volumes (**D**). Summary of volume data of tumors harvested after euthanizing the mice (**E**). Images of tumors harvested after euthanizing the mice (**F**). Representative images of HE and immunohistochemical staining of Keratin 7 expression in the lymph nodes harvested from mice treated with gefitinib (**G**). **H** A model of the c-Jun/CNOT3 feedback loop dysregulation contributes to gefitinib resistance and tumor progression. Data are shown as the mean±S.E.M. *n* = 4. **B** Student’s *t*-test. **D** Two-way ANOVA and Bonferroni post hoc test. **E** One-way ANOVA with Tukey post hoc test. ^***^P < 0.001, ^**^P < 0.01 or ^*^P < 0.05 for comparisons between the indicated groups.

To test whether depleting CNOT3 would overcome gefitinib resistance in vivo, a xenograft mouse model was established by subcutaneously injecting PC-9 GR2 cells which had been transfected with lentivirus harboring CNOT3-specific shRNA Tet-on inducible plasmid vectors into BALB/c nude mice (Fig. 6A, B). When the average tumor volume reached 50 mm^3^, gefitinib was given daily at 20 mg/kg by oral gavage, after which tumor volume was monitored every three days (Fig. 6C). We demonstrated CNOT3 depletion was able to inhibit tumor growth in vivo. In addition, we noticed the tumor growth was further inhibited by gefitinib and DOX co-treatment, suggesting CNOT3 depletion enhanced the anti-tumor effect of gefitinib (Fig. 6D–F). During our treatment, no significant difference in the body weight was observed between the four groups (Supplementary Fig. 6A). Unexpectedly, we noticed that mice in the gefitinib group had axillary fossa lymphadenectasis, which was neither observed in the co-treatment group, nor in other groups (Fig. 6G and Supplementary Fig. 6B). We performed immunohistochemistry to detect Keratin 7, a protein mainly expressed in epithelial cells and highly expressed in lung adenocarcinoma, thus to determine whether the lymphadenectasis was a result of tumor metastasis. The results showed there were some Keratin 7-positive cells in the lymph node, suggesting gefitinib treatment adversely triggered the metastasis of some drug-resistant tumor cells and CNOT3 depletion prevented it (Fig. 6G).

Collectively, these findings suggested that CNOT3 could be a potential target to overcome gefitinib resistance as well as metastatic progression.

## Discussion

CCR4-NOT complex is important for mammalian as it regulates gene expression in multiple processes such as chromatin remodeling, transcription and mRNA degradation [31]. Increasing reports have delineate the functions of each subunit and changes in their expressions may correlate with cancer malignancy [32–34]. However, till now few studies illustrate how their expressions are regulated. Our previous study has proposed the up-regulation of CNOT3 in lung cancer [22]. In this study, after consulting the TCGA database, we raised some possible molecules that would regulate CNOT3 expression and demonstrated here the expression of CNOT3 could be modulated by EGFR signaling. *EGFR* alterations (mutations, amplification) are commonly seen in lung cancer cells [35]. Our findings may also give an explanation on the overexpression of CNOT3 in lung cancer.

Evidences have shown that CNOT3 influences gene expression at all steps, from transcription to translation [12, 14]. We noticed that the mRNA level of c-Jun was not impacted by CNOT3 depletion, which suggested that the reduction in c-Jun protein expression did not result from an interference in transcription or a promotion in mRNA decay. It has been verified that in yeast harboring *not*5 (homolog to CNOT3) mutants, the newly produced proteins aggregate massively, which suggests defects in protein folding or in protein interactions of newly produced proteins during translation [36]. Consistently, our data showed an aggregation of c-Jun in ER when CNOT3 was depleted. In addition, we found that when CNOT3 was knocked down, the c-Jun protein was less stable and more c-Jun degraded through the ubiquitin-proteasome pathway, which suggested the ERAD was triggered in cells to clear the inaccurately translated c-Jun protein. Hence, our findings may not only explain how c-Jun expression is regulated by CNOT3, but also provide an example in mammalian that CNOT3 regulates gene expression during translation.

A previous study has suggested that the high basal level of c-Jun confers resistance to EGFR-TKIs in NSCLC and its up-regulation is caused by HIF-1α [25]. In our study, we noticed the c-Jun/CNOT3 axis was dysregulated in PC-9 GR cells, which consequently led to aberrant c-Jun and CNOT3 expression. However, we attributed this at least partially to the overexpression of HER2. *HER2* amplification is a mechanism of drug resistance shared in all generations of EGFR-TKIs [10, 37]. Amplification of *HER2* may lead to overexpression of its protein and activation of bypass signaling pathway, which weakens the ongoing target therapy to control tumor [38]. Based on our results, we assumed in PC-9 GR cells, the c-Jun/CNOT3 axis was modulated not only by EGFR but also by the overexpressed HER2. And that is why single application of gefitinib or tucatinib was unable to disrupt the dysregulated c-Jun/CNOT3 axis, and only when cells were treated with both tucatinib and gefitinib, CNOT3 expression was down-regulated. Thus, it seems the drug-resistant cells will take advantage of the HER2/c-Jun/CNOT3 axis to sustain cell survival and proliferation when EGFR is blocked by gefitinib.

To date, numerous studies have tried to find out solutions to EGFR-TKIs resistance. Some are successfully applied in clinical treatment such as Osimertinib, but most of them are less useful [39–42]. In this study, we discussed about whether CNOT3 would be a potential target to overcome gefitinib resistance as its expression was negatively correlated with the anti-tumor efficacy of gefitinib. Our data showed that CNOT3 might be at the intersection to steer both drug resistance and cell invasion, though how cell invasion is controlled by CNOT3 still needs to be uncovered.

Taken together, our study delineates a c-Jun/CNOT3 feedback loop and verified the role of CNOT3 in EGFR signaling. We propose that the dysregulated c-Jun/CNOT3 axis can contribute to EGFR-TKIs resistance and CNOT3 is a potential target to enhance the chemosensitivity of lung cancer cells to EGFR-TKIs and to prevent metastatic progression (Fig. 6H).

## Materials and methods

### Reagents and antibodies

Gefitinib, tucatinib and MG132 were obtained from MedChemExpress (HY-50895, HY-16069 and HY-13259). Actinomycin D was obtained from Amresco (J608). SP600125 and human EGF were purchased from Sigma-Aldrich (S5567 and E9644). PD153035 was purchased from Selleck (S6546). Cycloheximide was obtained from Beyotime (S1560).

The antibodies against human c-Jun, PARP-1, phosphorylated ERK1/2 (Thr202/Tyr204), phosphorylated mTOR (Ser2448), TSC1, JNK and phosphorylated JNK were from Cell Signaling Technology (9165, 9532, 4370, 5536, 6935, 9252 and 4668). The anti-CNOT3 antibody was purchased from Proteintech (11135-1-AP). The antibodies against phosphorylated c-Jun (S73), ki67 and ubiquitin were from Abcam (ab30620, Ab16667 and ab134953). The antibodies against ERK1/2 were purchased from Abcam (ab54230) and Cell Signaling Technology (4695). The antibody against mTOR was purchased from Santa Cruz Biotechnology (sc-517464). The antibody against Keratin 7 was obtained from ZSGB-BIO (ZM-0071).

### Cell culture and cell death induction

HCC827, A549 and NCI-H1975 cells were purchased from American Type Culture Collection. PC-9 cells were purchased from National Collection of Authenticated Cell Cultures. All cell lines have been authenticated by DNA STR profiling by our lab in the past three years. Mycoplasma-free cells were used to perform the experiments.

Cells were cultured in RPMI-1640 supplemented with 10% FBS, 2 mM L-glutamine, and 100 U/ml penicillin/streptomycin, and maintained at 37 °C in a 5% CO_2_ atmosphere. PC-9 GR1 and PC-9 GR2 cells were established in our lab by continuously exposure to increasing concentrations of gefitinib for 12 months, and PC-9 cells were used as the parent cells. To induce death, HCC827, PC-9, PC-9 GR1 and PC-9 GR2 cells were treated with 1 μM gefitinib for 24 h.

### Cell survival and cell proliferation assay

Cell viability was measured by Cell Counting Kit 8 (CCK-8) purchased from TargetMol (C0005). After drug treatment, a 10 μl CCK-8 solution was added to the culture medium of cells growing in 96-well plates (5000 cells/well) and incubated in a humidified CO_2_ incubator at 37 °C for 2 h. Then, samples were measured at 450 nm and the data was normalized to background readings of media only.

To perform proliferation assay, cells were seeded in 96-well plates (1,000 cells/well) after transfection and allowed to adhere. A 10 μl CCK-8 solution was added to each well at the indicated times and then measured absorbance at 450 nm.

### Recombinant plasmid construction and transfection

The full length human CNOT3-EGFP recombinant plasmid was constructed as previously described [22]. The PC-9 cell line with stable transfection of the vector or the CNOT3-EGFP recombinant plasmid were screened by G418 and flow cytometry.

A series of pGL3 reporter plasmids were obtained from BGI. Cells with 80% confluence were transfected using LipofectamineTM 2000 reagent (11668019, Invitrogen Life Technologies) and the experimental procedure was carried out according to the manufacturer’s instructions. Forty-eight hours after transfection, cells were used to performed the sequential assays.

Lentivirus harboring CNOT3-specific shRNA Tet-on inducible plasmid vector was constructed by and purchased from GENECHEM (GV307).

### RNA interference

The siRNAs for human CNOT3, c-Jun, TSC1 and the non-target siRNA were designed by and obtained from GenePharma.

CNOT3 siRNA^#1^: 5′-GGACAAGCGCAAACUCCAATT-3′ and 5’-UUGGAGUUUGCGCUUGUCCTT-3′;

CNOT3 siRNA^#2^: 5′-GCUGUCAGAGGUGAACAUATT-3′ and 5′-UAUGUUCACCUCUGACAGCTT-3′.

c-Jun siRNA^#1^: 5′-GGCACAGCUUAAACAGAAATT-3′ and 5′-UUUCUGUUUAAGCUGUGCCTT-3′.

c-Jun siRNA^#2^: 5′-CGAUCUCAUUCAGUAUUAATT-3′ and 5′-UUAAUACUGAAUGAGAUCGTT-3′.

TSC1 siRNA^#2^: 5′-CCAAAUCUCAGCCCGCUUUTT-3′ and 5′- AAAGCGGGCUGAGAUUUGGTT-3′.

TSC1 siRNA^#3^: 5′-GCAAGCCUUUACUCCAUATT-3′ and 5′-UAUGGGAGUAAAGGCUUGCTT-3′.

non-target siRNA: 5′-UUCUCCGAACGUGUCACGUTT-3’ and 5′-ACGUGACACGUUCGGAGAATT-3′.

Cells with 50% confluence were transfected with the siRNAs (100 pmol) using the Lipofectamine^TM^ 2000 reagent according to the manufacturer’s instructions. Twenty-four hours after transfection, cells were used to performed the sequential assays.

### Flow cytometry analysis

Annexin V-APC/7-AAD Apoptosis Detection Kit was obtained from Keygen Biotech (KGA1025) and Elabscience (E-CK-A218). Cell death was recorded on a FACSCalibur flow cytometer (Becton, Dickinson and Company) in the total population (10,000 cells), and the data were analyzed using FlowJo software (Version 7.6.1).

### Quantitative PCR (q–PCR) analysis

To perform the analysis, cells were collected and Total RNA was isolated with the E.Z.N.A.® Total RNA Kit II (Omega Bio-tek) according to the manufacturer’s protocol. cDNAs were synthesized using PrimeScript™ RT Master Mix (Perfect Real Time) (RR036A, Takara) as previously described [22]. Quantitative PCR analysis was performed using the TB Green® Premix Ex Taq™ II (Tli RNaseH Plus) (RR820A, Takara) as previously described [22]. To measure mRNA stability, cells were treated with Actinomycin D (5 μg/ml), and total RNA was extracted at the indicated times and subjected to q-PCR analysis.

Human CNOT3 forward: 5′-GGACGTTCCACAGACAGTGA-3′; reverse: 5′-GAGGGTGCTGGTTGCTGT-3′.

Human c-Jun forward: 5′-TCCAAGTGCCGAAAAAGGAAG-3′; reverse: 5′-CGAGTTCTGAGCTTTCAAGGT-3′.

Human TSC1 forward: 5′-CAACAGGCGTCTTGGTGTTG-3′; reverse: 5′-ACACACTGGCATGGAGATGG-3′.

Human GAPDH forward: 5′-GCACCGTCAAGGCTGAGAAC-3′; reverse: 5′-TGGTGAAGACGCCAGTGGA-3′.

### Western blotting and immunoprecipitation

For western blotting, cells were collected after treatment. RIPA lysis buffer obtained from Beyotime Institute of Biotechnology (P0013B) was added to generate cell lysates. The protein concentrations of the samples were determined with a Pierce™ BCA Protein Assay Kit (23225, Thermo Fisher Scientific) and then equalized. An equal volume of each cell lysate was resolved via 10% SDS-PAGE, transferred onto Immobilon-P membranes (Millipore), and then incubated 4 °C overnight with primary antibodies diluted 1:1000 with PBS-T buffer (136 mM NaCl, 2.6 mM KCl, 8 mM Na_2_HPO_4_, 2 mM KH_2_PO_4_, 0.05% Tween-20) followed by incubation with an HRP-conjugated secondary antibody (1:4000) for 1 h.

To perform immunoprecipitation, about 10,000,000 cells were dissociated and collected. The Pierce™ Co-Immunoprecipitation Kit (26149, Thermo Fisher Scientific) was used, and all of the experimental procedures were performed according to the manufacturer’s instructions.

### Immunocytochemistry

PC-9 cells with or without CNOT3 depletion were seeded in 35 mm culture dishes. Then, the cells were treated with MG132 (1 μM) or left untreated. After that, the cells were fixed in 4% paraformaldehyde for 15 min, washed with PBS and permeabilized with 0.2% Triton X-100 for 15 min at room temperature. Next, the cells were washed again with PBS and incubated in 1% goat serum albumin for 1 h at room temperature. The cells were then incubated with an antibody against c-Jun overnight at 4 °C, and this was followed by incubation with fluorescein-conjugated secondary antibody for 1 h at room temperature. During incubation with the secondary antibody, the endoplasmic reticulum was stained with ER-Tracker™ Green (E34251, Invitrogen Life Technologies). The nuclei were stained with DAPI. At last, the samples were examined under an Olympus FV10i-LIV microscope (Olympus, Tokyo, Japan).

### Dual-Luciferase reporter assay

Recombinant pGL3 reporter plasmids and pRL-TK vectors were co-transfected into PC-9 and HCC827 cells with or without c-Jun depletion. The Dual-luciferase Reporter Assay (E1910, Promega) was performed according to the manufacturer’s instructions.

### Tumor xenografts

All animal experiments were performed with the approval of the Institutional Animal Care and Use Committee (IACUC) of the Fourth Military Medical University. Male BALB/c nude mice (4 weeks of age and weighing 16–20 g) were purchased from Beijing Vital River Laboratory Animal Technologies and were acclimatized for 1 week. The mice were maintained in a specific pathogen-free environment and were given free access to standard chow and water. To induce the expression of CNOT3-shRNA, food containing 100 mg/kg doxycycline (DOX) was used to feed mice. PC-9 GR2 cells (1,000,000) stably transfected with CNOT3-shRNA were subcutaneously inoculated into the mice to establish a lung adenocarcinoma model. When the average tumor volume reached 50 mm^3^, mice were randomly distributed into groups of 4 mice. Mice were received either vehicle control, gefitinib alone, DOX alone or gefitinib and DOX together. Gefitinib was dissolved in solvent containing 10% DMSO, 40% PEG300, 5% Tween-80 and 45% saline, and administered every day by oral gavage (20 mg/kg). Every three days, tumor sizes were measured by a digital caliper. Tumor volume was calculated according to the formula: V = (a × b^2^)/2, where a and b are the maximal and minimal diameters in millimeters, respectively. Every five days, the mice were weighed. Eighteen days later, the mice were sacrificed and tumors were immediately weighed.

### Immunohistochemistry

Strict serial 4 μm sections were cut from formalin-fixed paraffin-embedded tissue blocks. The detection of Keratin 7-positive cells was performed via staining the sections with a primary antibody against Keratin 7 using SP kit (SP-9002, ZSGB-BIO) according to the manufacturer’s instructions. Stains were detected using anti-immunoglobulin-coupled horseradish peroxidase with 3,3′-diaminobenzidine (DAB, ZLI-9018, ZSGB-BIO) as substrate. Nuclear counterstaining was performed with Cole hematoxylin (G1140, Solarbio).

### Statistical analysis

Data are expressed as mean ±S.E.M. of three independent experiments. Statistical analysis was performed using Student’s *t*-test, Mann–whitney U test or ANOVA. Spearman correlation test was used to analyze the correlation of the parameters in the two groups and the degree of correlation was expressed by Spearman correlation coefficient “r”. The GraphPad Prism 5.0 program was used for the statistical analysis.

### Supplementary information

Supplementary Figure 1

Supplementary Figure 2

Supplementary Figure 3

Supplementary Figure 4

Supplementary Figure 5

Supplementary Figure 6

Supplementary Figure 7

supplementary figure legend

western blotting uncropped

## Data Availability

The data and materials are available by contacting the corresponding author upon reasonable request.
