# Supplementary figures and images for: Targeting the up-regulated CNOT3 reverses therapeutic resistance and metastatic progression of EGFR-mutant non-small cell lung cancer
Source: Cell Death Discov. 2023 Nov 2;9:406. doi: 10.1038/s41420-023-01701-w (PMC10622567; doi:10.1038/s41420-023-01701-w)

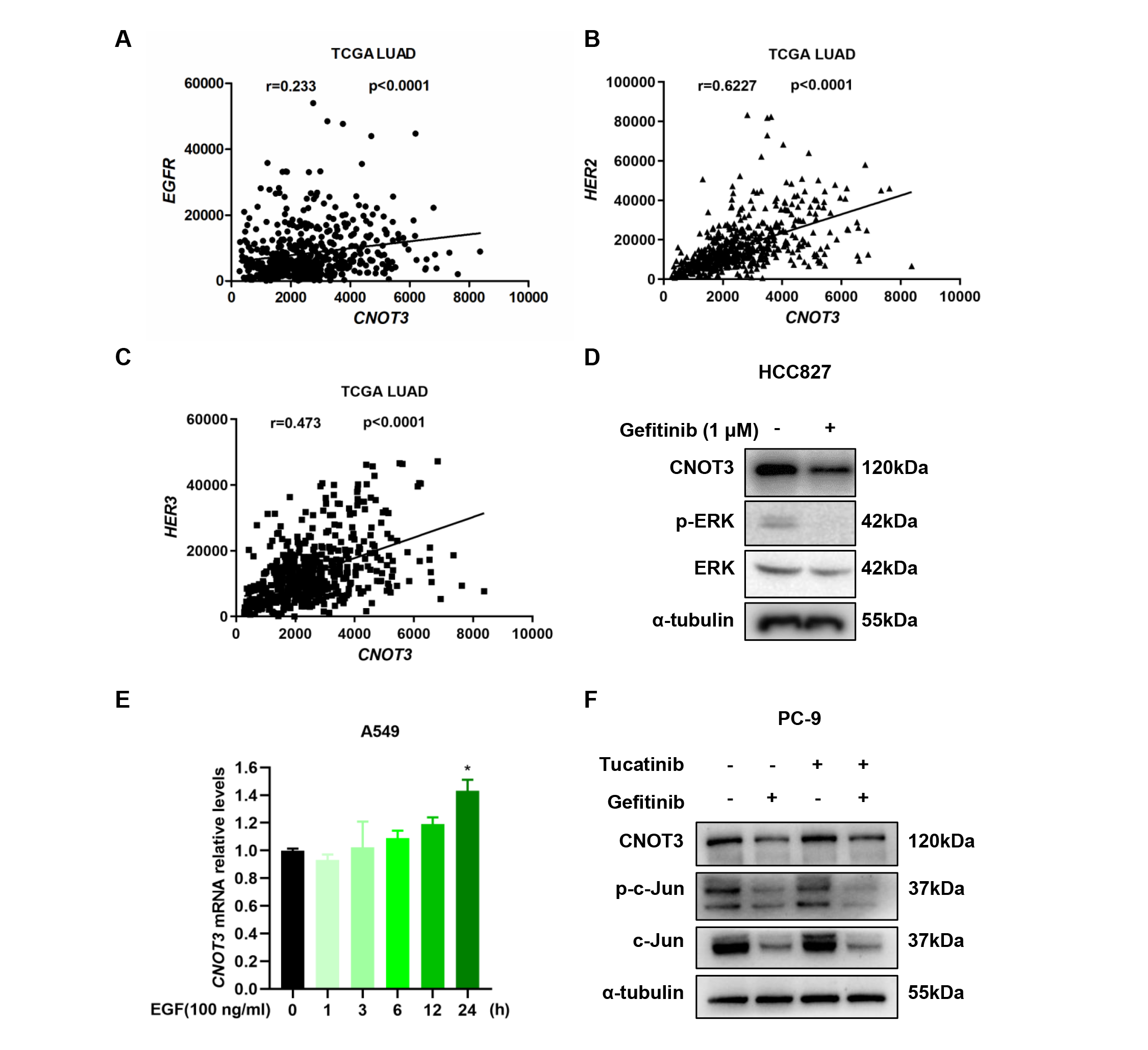

Supplement: Supplementary file 1 — Supplementary Figure 1 [file 41420_2023_1701_MOESM1_ESM.tif]

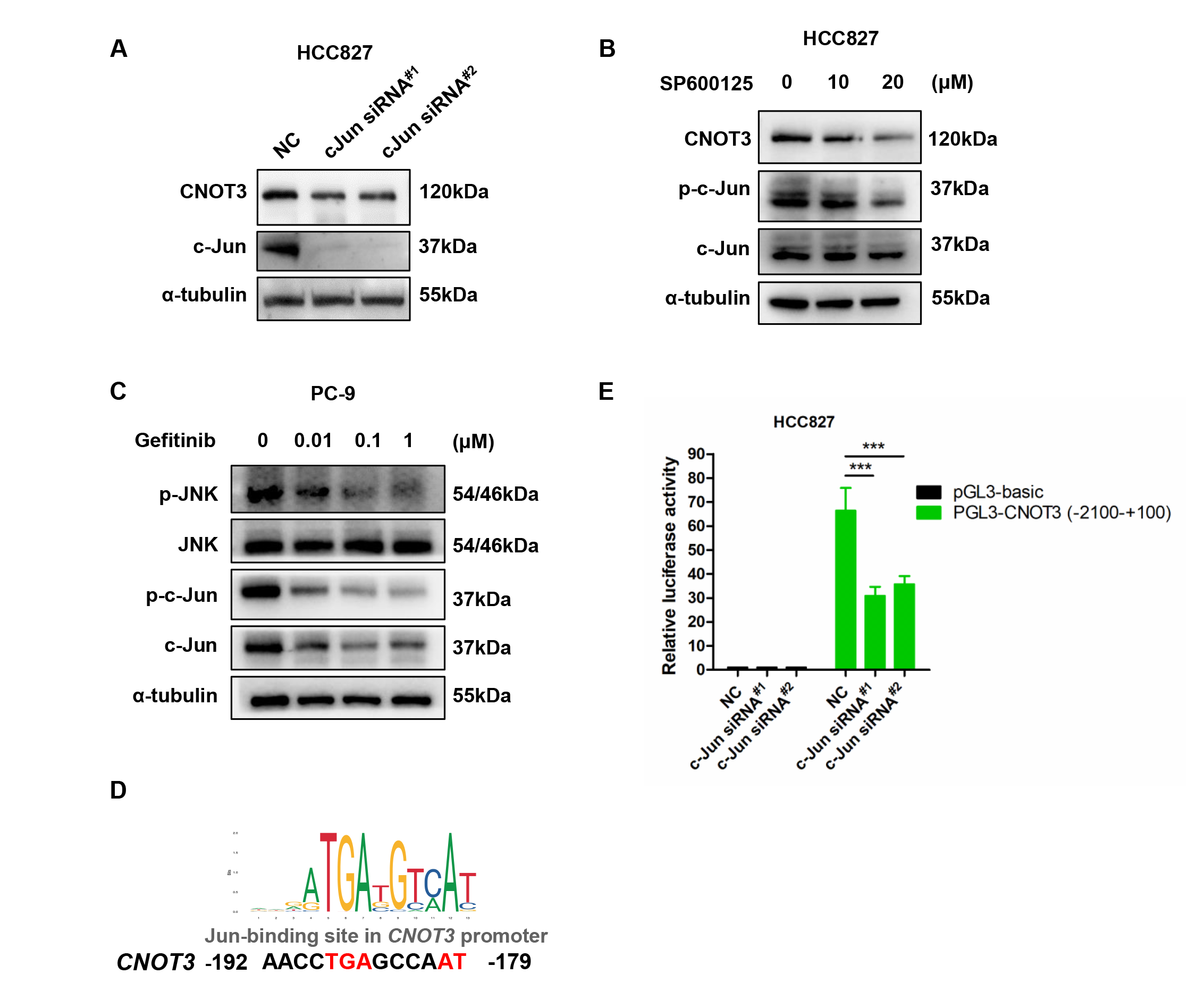

Supplement: Supplementary file 2 — Supplementary Figure 2 [file 41420_2023_1701_MOESM2_ESM.tif]

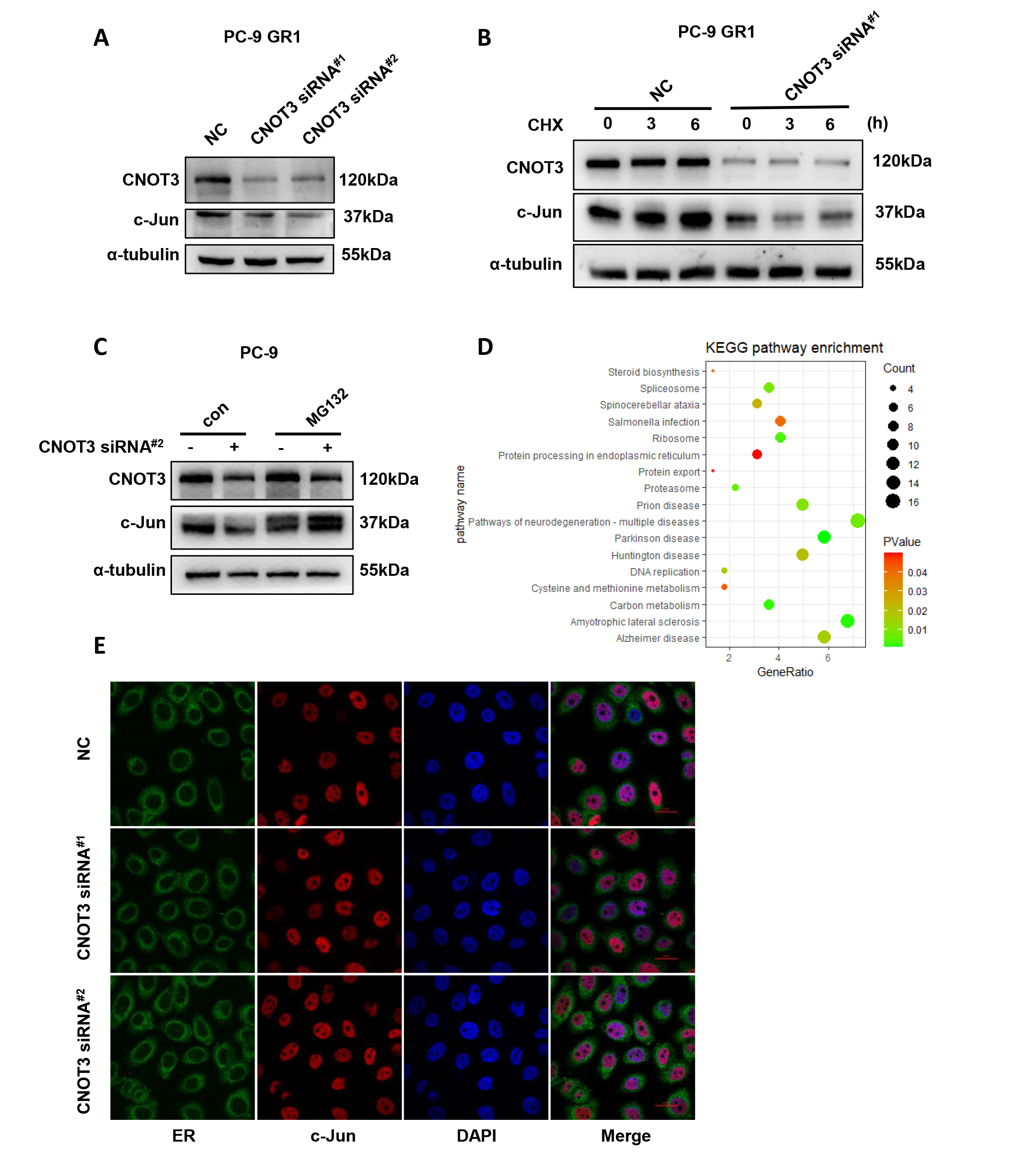

Supplement: Supplementary file 3 — Supplementary Figure 3 [file 41420_2023_1701_MOESM3_ESM.tif]

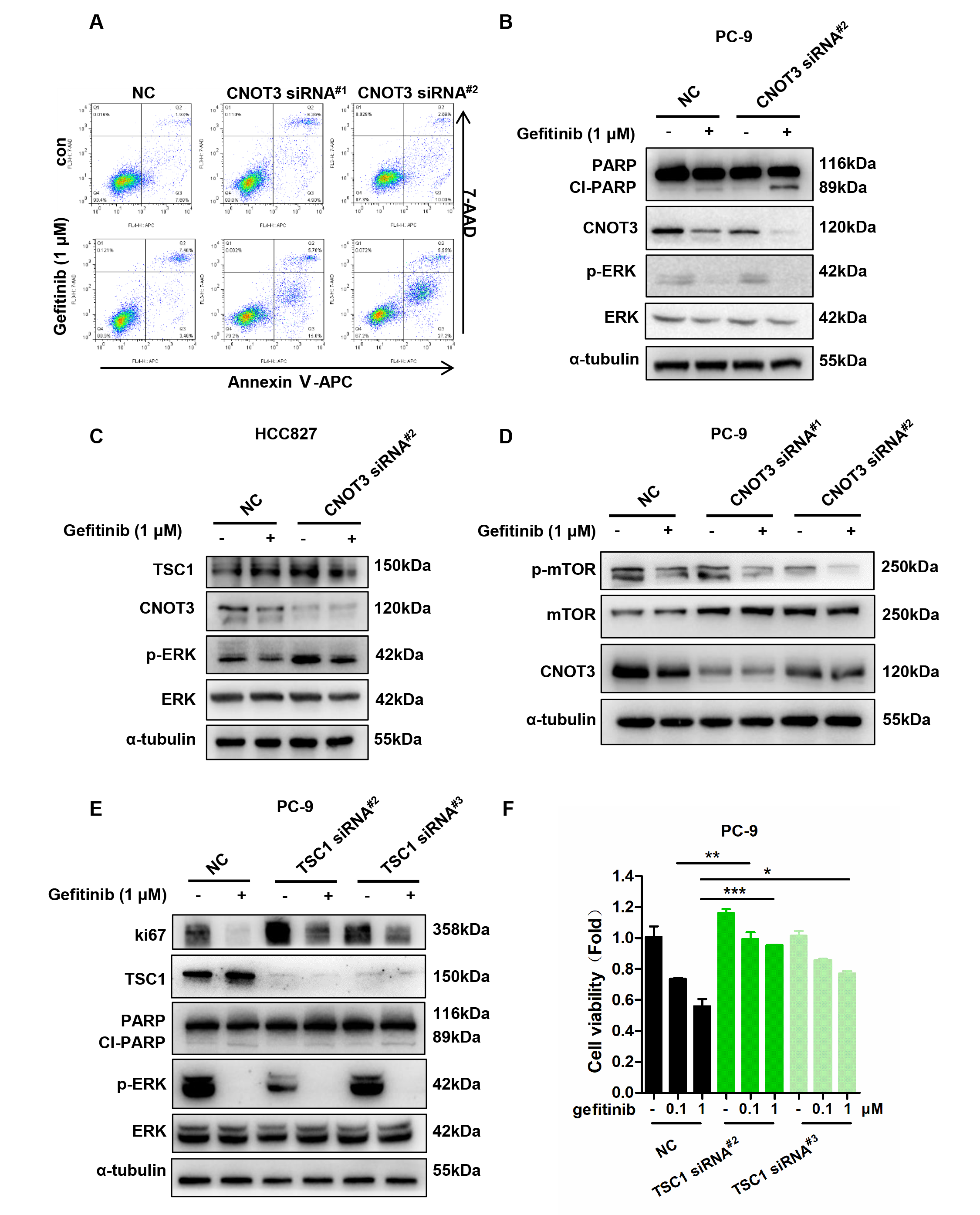

Supplement: Supplementary file 4 — Supplementary Figure 4 [file 41420_2023_1701_MOESM4_ESM.tif]

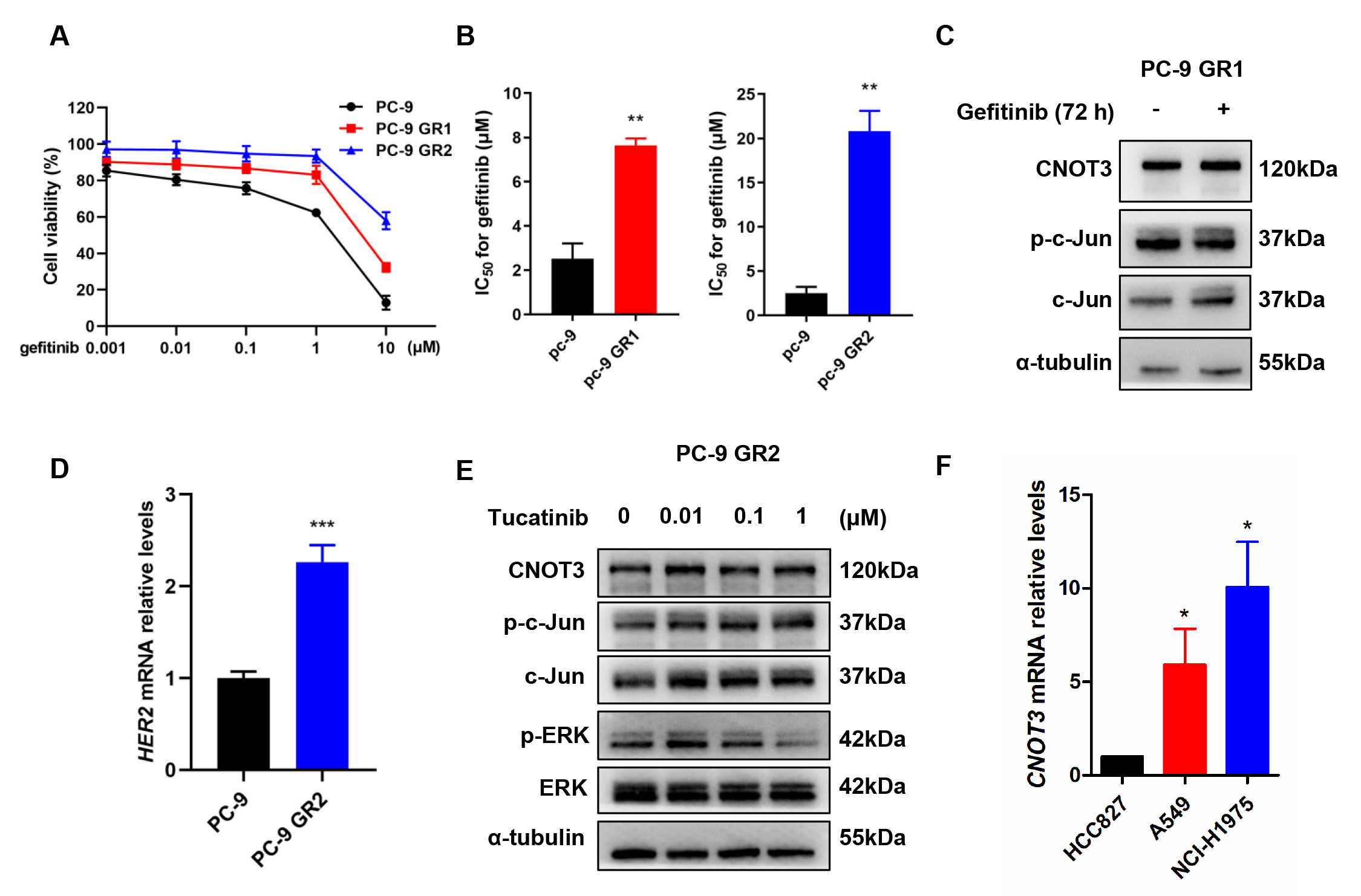

Supplement: Supplementary file 5 — Supplementary Figure 5 [file 41420_2023_1701_MOESM5_ESM.tif]

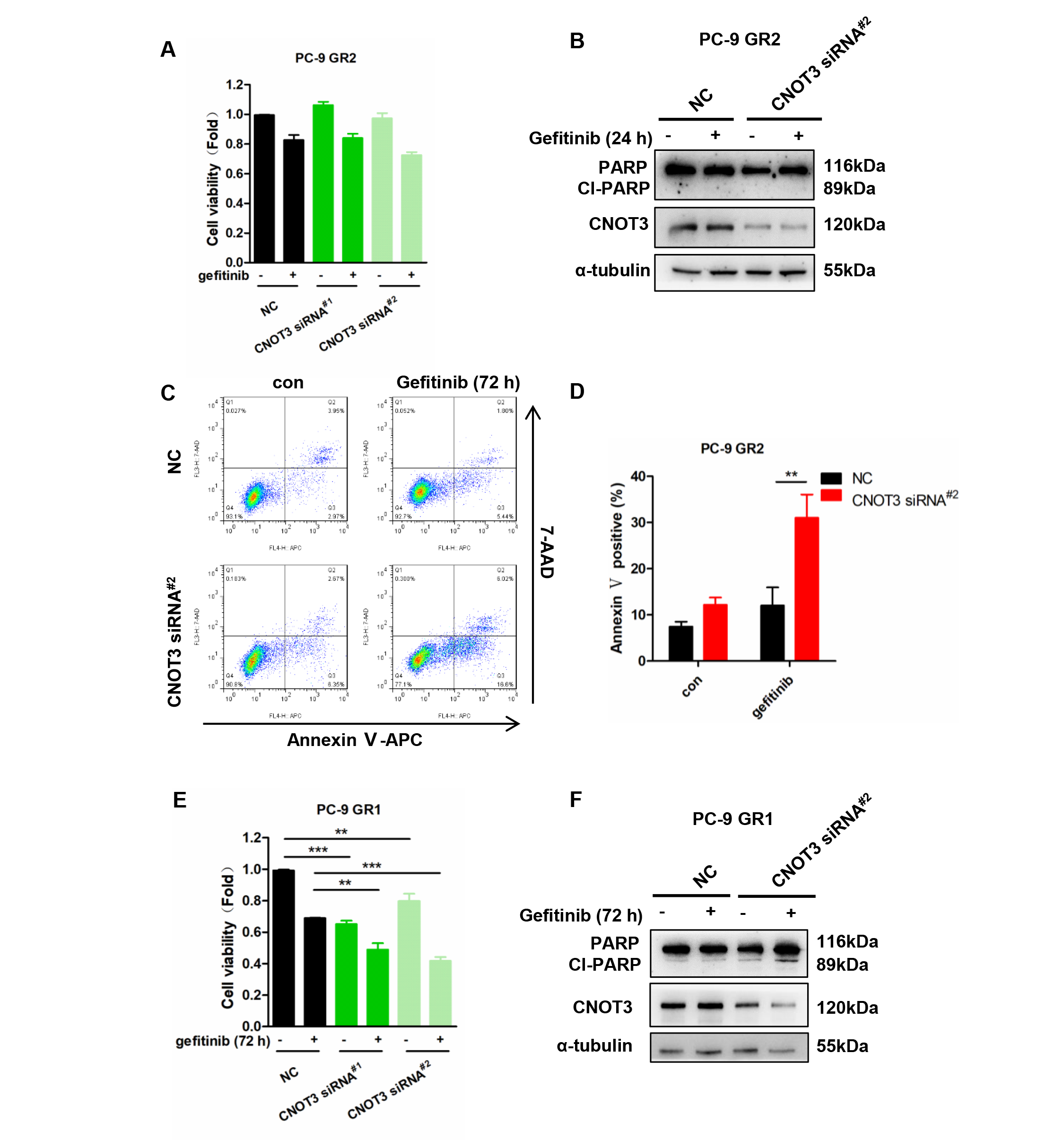

Supplement: Supplementary file 6 — Supplementary Figure 6 [file 41420_2023_1701_MOESM6_ESM.tif]

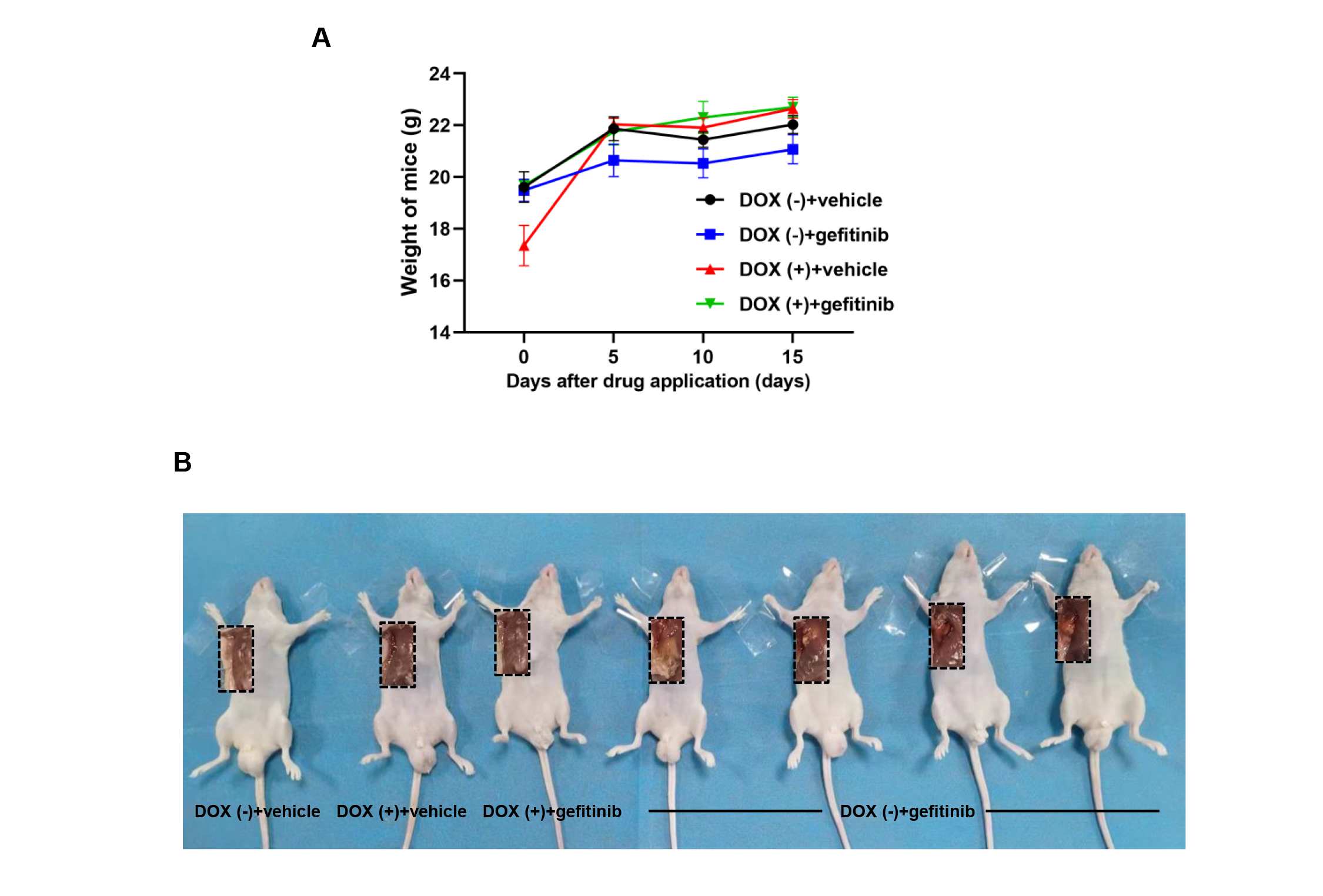

Supplement: Supplementary file 7 — Supplementary Figure 7 [file 41420_2023_1701_MOESM7_ESM.tif]
