# Supplementary material for: Targeting the up-regulated CNOT3 reverses therapeutic resistance and metastatic progression of EGFR-mutant non-small cell lung cancer
Source: Cell Death Discov. 2023 Nov 2;9:406. doi: 10.1038/s41420-023-01701-w (PMC10622567; doi:10.1038/s41420-023-01701-w)
Supplement: Supplementary file 8 — supplementary figure legend [file 41420_2023_1701_MOESM8_ESM.docx]

**Supplementary Materials**

**Supplementary Figure 1**

**EGFR signaling regulates CNOT3 expression.** **A-C** *CNOT3* expression levels were positively related with *EGFR* (A), *HER2* (B) and *HER3* (C) expression in lung adenocarcinoma tissues. Data was downloaded from TCGA database. n=522, 528 and 520 respectively. **D** HCC827 cells were treated with gefitinib for 24 hours, then cells were subjected to western blotting to assess protein expression levels. **E** A549 cells were treated with human EGF for the indicated time after serum starvation for 12 h, *CNOT3* mRNA levels in cells were measured by q-PCR. **F** PC-9 cells were treated with tucatinib (1 μM) and/or gefitinib (1 μM) for 24 h, then cells were subjected to western blotting to assess protein expression levels. Data are shown as the mean±S.E.M.. n=3. **E** One-way ANOVA with Tukey post hoc test. ^*^*P* < 0.05 for comparisons between the indicated groups.

**Supplementary Figure 2**

**CNOT3 is transcriptionally regulated by c-Jun. A** HCC827 cells were transfected with a control or c-Jun siRNA, then cells were subjected to western blotting to assess protein expression levels. **B** HCC827 cells were treated with SP600125 for the indicated time, then cells were subjected to western blotting to assess protein expression levels. **C** PC-9 cells were treated with gefitinib for 24 hours, then cells were subjected to western blotting to assess protein expression levels. **D** The predicted binding sequences of c-Jun to CNOT3. **E** HCC827 cells were transfected with c-Jun siRNA and luciferase reporter plasmids containing *CNOT3* promoter, followed by luciferase reporter assays. Data are shown as the mean±S.E.M.. n=3. **E** Two-way ANOVA and Bonferroni post hoc test. ^***^*P* < 0.001 for comparisons between the indicated groups.

**Supplementary Figure 3**

**A feedback loop exists between c-Jun and CNOT3.** **A** PC-9 GR1 cells were transfected with a control or CNOT3 siRNA. Forty-eight hours after transfection, cells were subjected to western blotting to assess protein expression levels. **B** PC-9 GR1 cells were transfected with a control or CNOT3 siRNA^#1^. Twenty-four hours after transfection, cells were treated with CHX (10 μM) for the indicated time, then cells were subjected to western blotting to assess protein expression levels. **C** PC-9 cells were transfected with a control or CNOT3 siRNA^#2^. Twenty-four hours after transfection, cells were treated with MG132 (1 μM) for 9 hours, then cells were subjected to western blotting to assess protein expression levels. **D** Genes that were up-regulated over 1.5-fold in the CNOT3-depleted HCC827 cells were identified using KEGG analysis. **E** PC-9 cells were transfected with a control or CNOT3 siRNA. Thirty-three hours after transfection, then PC-9 cells were stained with anti-c-Jun antibody, ER tracker and DAPI.

**Supplementary Figure 4**

**CNOT3 regulates cell proliferation via influencing TSC1/mTOR axis. A** Representative images of cell death analyzed by the flow cytometry. **B-D** PC-9 (B and D) or HCC827 (C) cells were transfected with a control or CNOT3 siRNA. Twenty-four hours after transfection, cells were treated with gefitinib for 24 h. Cells were subjected to western blotting to assess protein expression levels. **E and F** PC-9 cells were transfected with a control or TSC1 siRNA. Twenty-four hours after transfection, cells were treated with gefitinib for 24 h. Cells were subjected to western blotting to assess protein expression levels (E). Cell viability was measured via the CCK-8 assay (F). Data are shown as the mean±S.E.M.. n=3. **F** One-way ANOVA with Tukey post hoc test. ^***^*P* < 0.001, ^**^*P* < 0.01 or ^*^*P* < 0.05 for comparisons between the indicated groups.

**Supplementary Figure 5**

**The c-Jun/CNOT3 axis is dysregulated in PC-9 GR cells. A and B** PC-9, PC-9 GR1 and PC-9 GR2 cells were treated with indicated doses of gefitinib for 24 h, and then cell viability and IC_50_ of gefitinib were determined using the CCK-8 assay. **C** PC-9 GR1 cells were treated with gefitinib for 72 h, then cells were subjected to western blotting to assess protein expression levels. **D** *HER2* mRNA levels in PC-9 and PC-9 GR2 cells were measured by q-PCR. **E** PC-9 GR2 cells were treated with tucatinib for 24 h, then cells were subjected to western blotting to assess protein expression levels. **F** *CNOT3* mRNA levels in HCC827, A549 and H1975 cells were measured by q-PCR. Data are shown as the mean±S.E.M.. n=3. **B and D** Student’s *t*-test. **F** One-way ANOVA with Tukey post hoc test. ^***^*P* < 0.001, ^**^*P* < 0.01 or ^*^*P* < 0.05 for comparisons between the indicated groups.

**Supplementary Figure 6**

**CNOT3 depletion enhances the anti-tumor effect of gefitinib in vitro. A and B** PC-9 GR2 cells were transfected with a control or CNOT3 siRNA. Twenty-four hours after transfection, cells were treated with gefitinib for 24 h. Cell viability was measured via the CCK-8 assay (A). Cells were subjected to western blotting to assess protein expression levels (B). **C and D** PC-9 GR2 cells were transfected with a control or CNOT3 siRNA^#2^. Twenty-four hours after transfection, cells were treated with gefitinib for 72 h. Cell death was analyzed via the flow cytometry. **E and F** PC-9 GR1 cells were transfected with a control or CNOT3 siRNA. Twenty-four hours after transfection, cells were treated with gefitinib for 72 h. Cell viability was measured via the CCK-8 assay (E). Cells were subjected to western blotting to assess protein expression levels (F). Data are shown as the mean±S.E.M.. n=3. **A and E** One-way ANOVA with Tukey post hoc test. **D** Two-way ANOVA and Bonferroni post hoc test. ^***^*P* < 0.001 or ^**^*P* < 0.01 for comparisons between the indicated groups.

**Supplementary Figure 7**

**Down-regulating CNOT3 overcomes gefitinib resistance and inhibits metastatic progression in vivo. A** Plots of body weight. **B** Anatomical images of mice without axillary fossa lymphadenectasis (three on the left) or with axillary fossa lymphadenectasis (four on the right). Data are shown as the mean±S.E.M.. n=4. **A** Two-way ANOVA and Bonferroni post hoc test.
