# Supplementary material for: Targeting the up-regulated CNOT3 reverses therapeutic resistance and metastatic progression of EGFR-mutant non-small cell lung cancer
Source: Cell Death Discov. 2023 Nov 2;9:406. doi: 10.1038/s41420-023-01701-w (PMC10622567; doi:10.1038/s41420-023-01701-w)

Figure 1

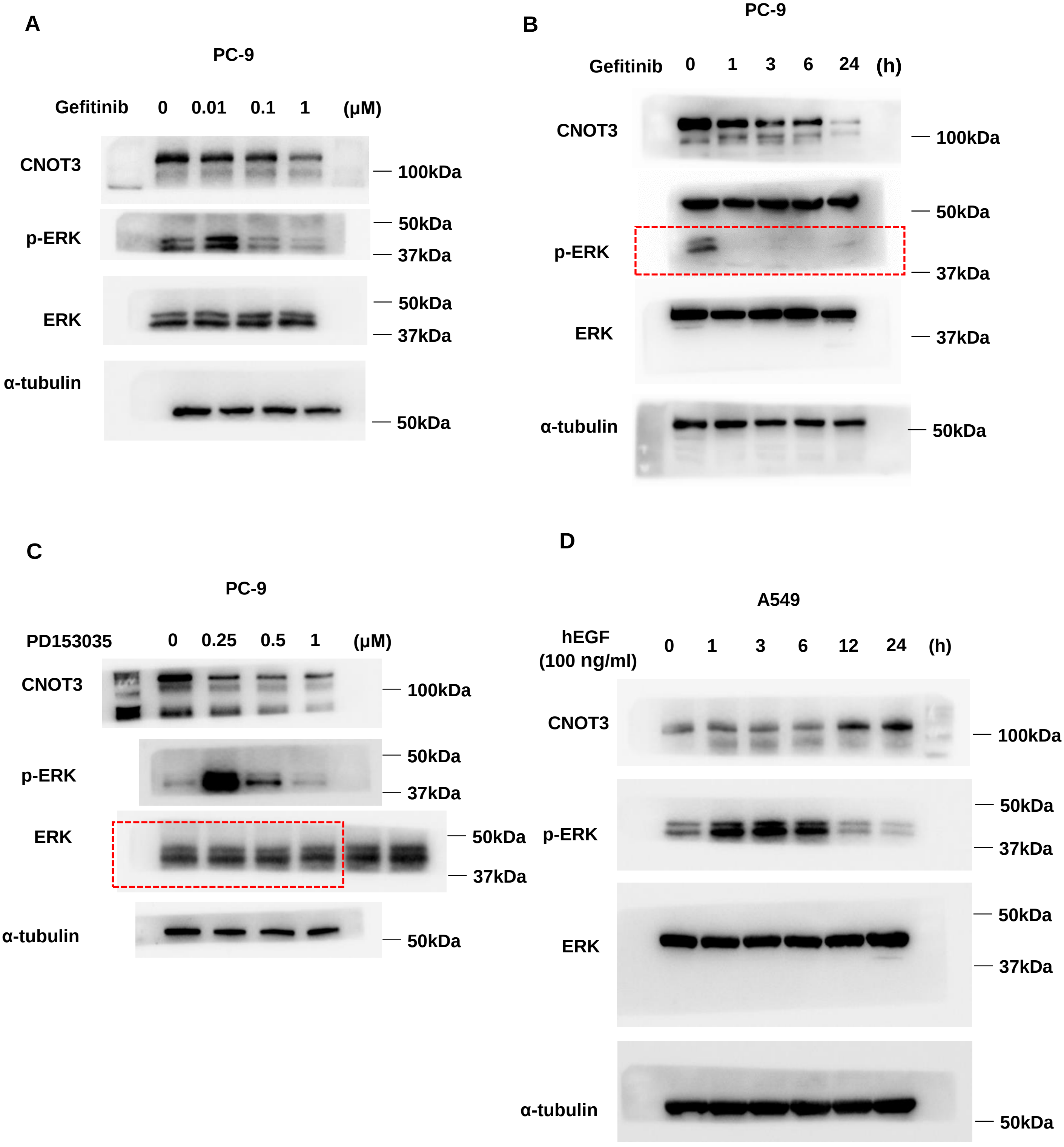
A
PC-9
Gefitinib 0 0.01 0.1 1 (μM)
CNOT3
p-ERK
ERK
α-tubulin
100kDa
50kDa
37kDa
50kDa
37kDa
50kDa
B
PC-9
Gefitinib 0 1 3 6 24 (h)
CNOT3
p-ERK
ERK
α-tubulin
100kDa
50kDa
37kDa
37kDa
50kDa
C
PC-9
PD153035 0 0.25 0.5 1 (μM)
CNOT3
p-ERK
ERK
α-tubulin
100kDa
50kDa
37kDa
50kDa
37kDa
50kDa
D
A549
hEGF (100 ng/ml) 0 1 3 6 12 24 (h)
CNOT3
p-ERK
ERK
α-tubulin
100kDa
50kDa
37kDa
50kDa
37kDa
50kDa

Figure 1

F

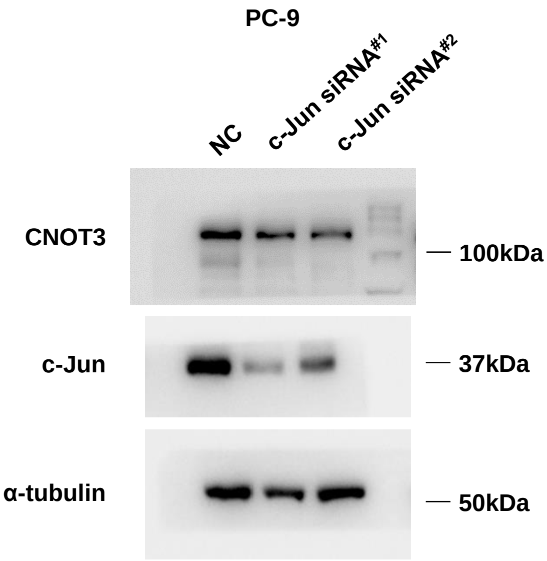

G

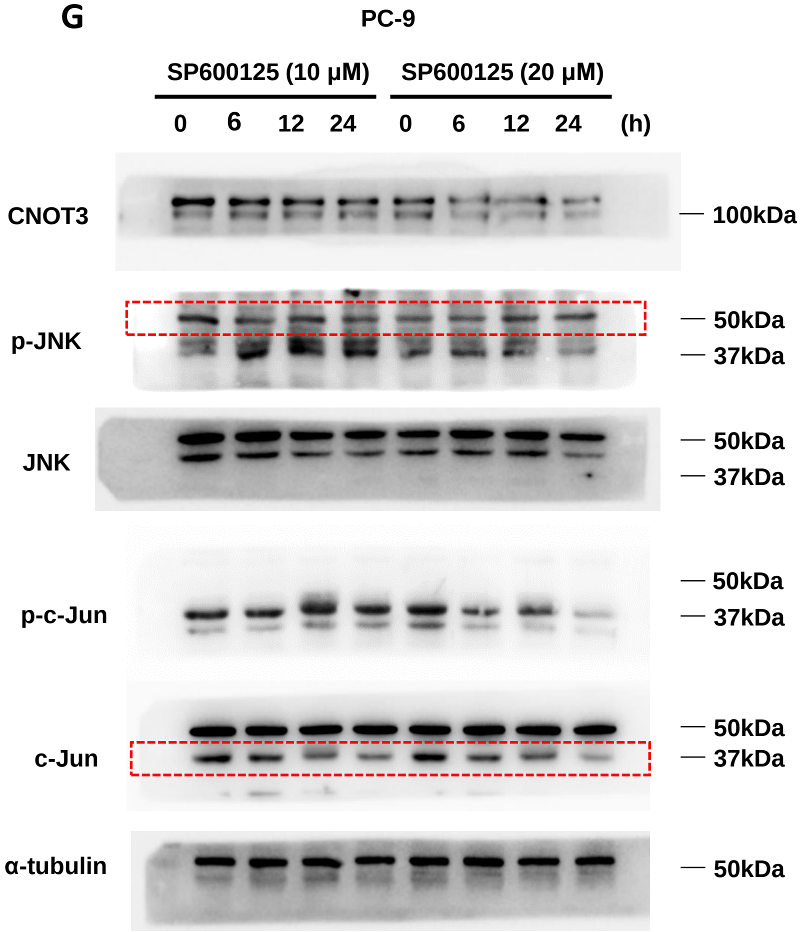

Supplementary Figure 1

D

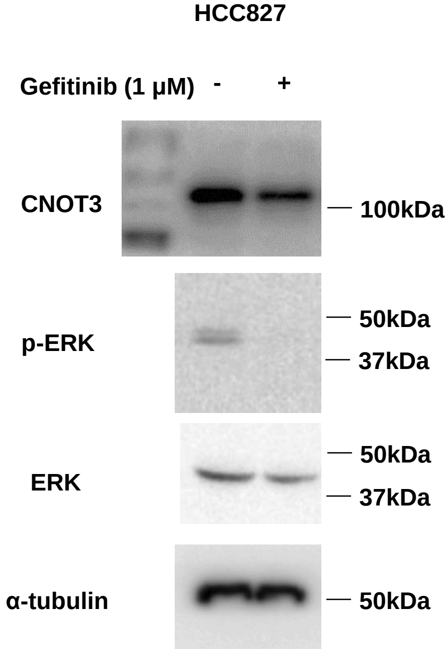

F

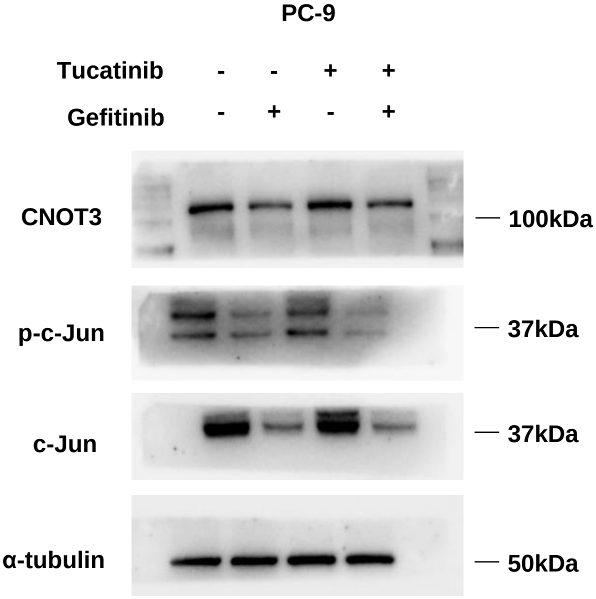

Figure 2

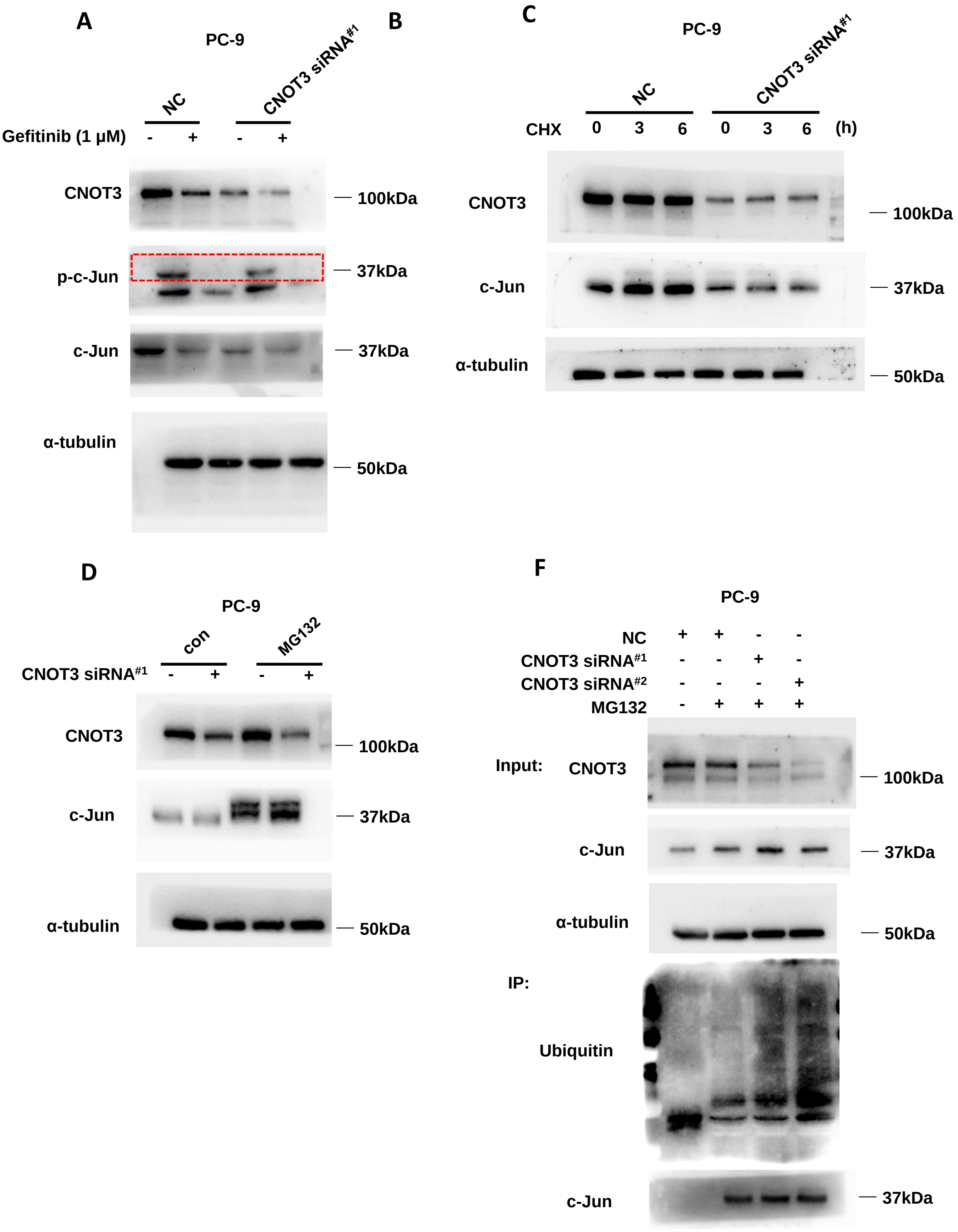
A
B
PC-9
NC
CNOT3 siRNA#1
Gefitinib (1 μM)
- + - +
CNOT3
100kDa
p-c-Jun
37kDa
c-Jun
37kDa
α-tubulin
50kDa
C
PC-9
NC
CNOT3 siRNA#1
CHX
0 3 6 0 3 6 (h)
CNOT3
100kDa
c-Jun
37kDa
α-tubulin
50kDa
D
PC-9
con
MG132
CNOT3 siRNA#1
- + - +
CNOT3
100kDa
c-Jun
37kDa
α-tubulin
50kDa
F
PC-9
NC + + - -
CNOT3 siRNA#1 - - + -
CNOT3 siRNA#2 - - - +
MG132 - + + +
Input:
CNOT3
100kDa
c-Jun
37kDa
α-tubulin
50kDa
IP:
Ubiquitin
c-Jun
37kDa

Figure 3

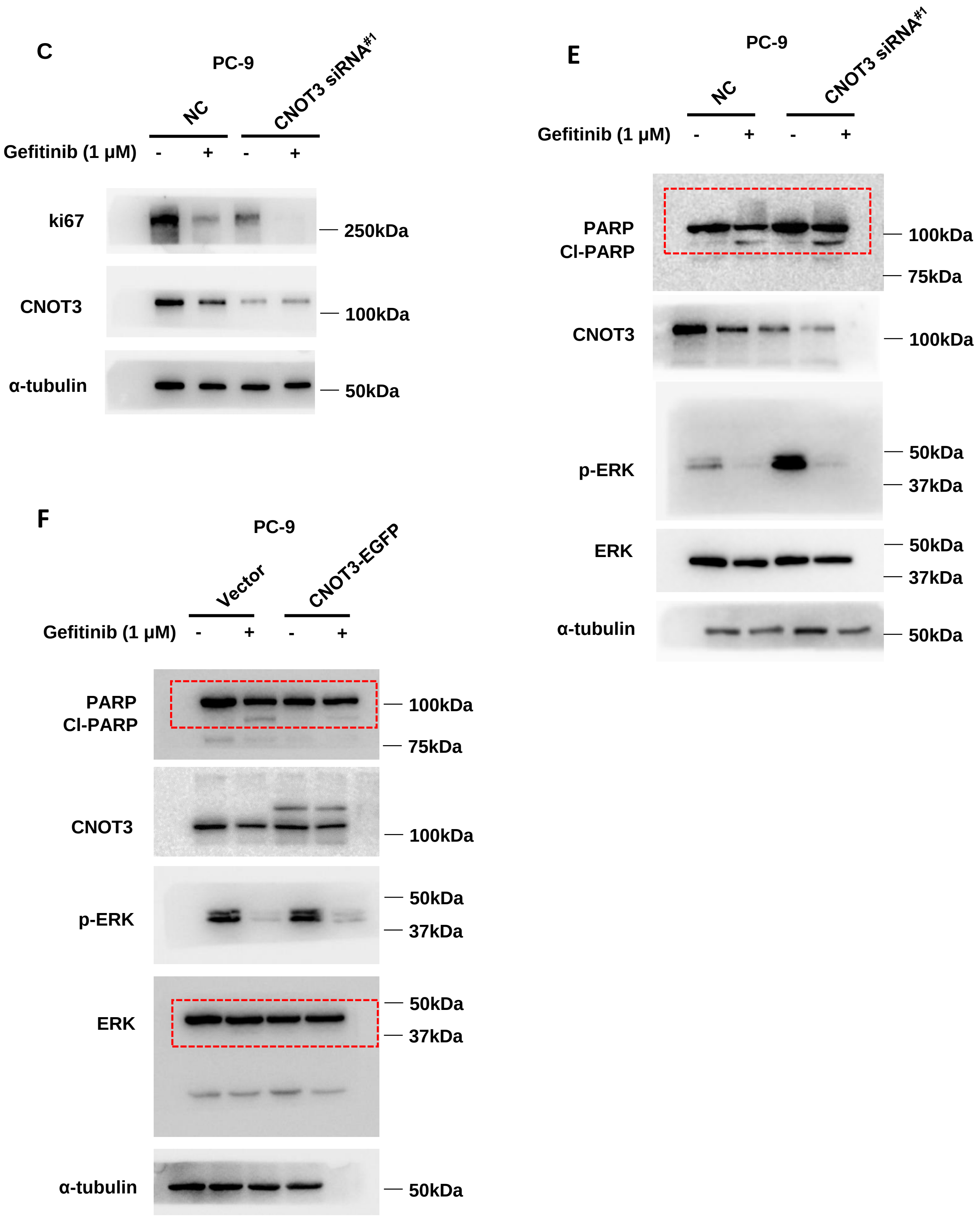

C
PC-9
NC
CNOT3 siRNA#1
Gefitinib (1 μM)
- + - +
ki67
250kDa
CNOT3
100kDa
α-tubulin
50kDa
E
PC-9
NC
CNOT3 siRNA#1
Gefitinib (1 μM)
- + - +
PARP
Cl-PARP
100kDa
75kDa
CNOT3
100kDa
p-ERK
50kDa
37kDa
ERK
50kDa
37kDa
α-tubulin
50kDa
F
PC-9
Vector
CNOT3-EGFP
Gefitinib (1 μM)
- + - +
PARP
Cl-PARP
100kDa
75kDa
CNOT3
100kDa
p-ERK
50kDa
37kDa
ERK
50kDa
37kDa
α-tubulin
50kDa

Figure 4

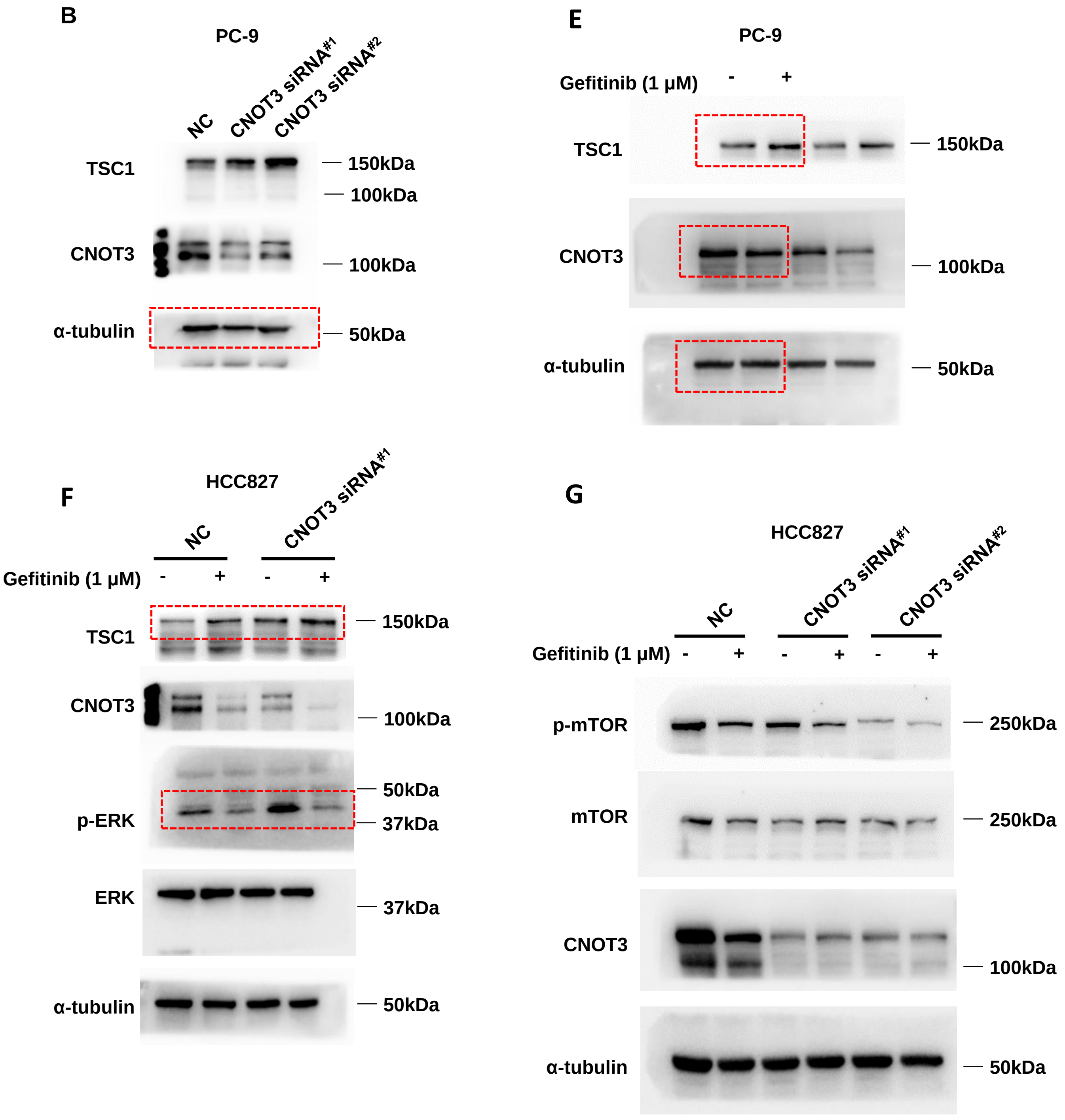

B
PC-9
NC
CNOT3 siRNA#1
CNOT3 siRNA#2
TSC1
150kDa
100kDa
CNOT3
100kDa
α-tubulin
50kDa
E
PC-9
Gefitinib (1 μM)
-
+
TSC1
150kDa
CNOT3
100kDa
α-tubulin
50kDa
F
HCC827
NC
CNOT3 siRNA#1
Gefitinib (1 μM)
-
+
-
+
TSC1
150kDa
CNOT3
100kDa
p-ERK
50kDa
37kDa
ERK
37kDa
α-tubulin
50kDa
G
HCC827
NC
CNOT3 siRNA#1
CNOT3 siRNA#2
Gefitinib (1 μM)
-
+
-
+
-
+
p-mTOR
250kDa
mTOR
250kDa
CNOT3
100kDa
α-tubulin
50kDa

Figure 5

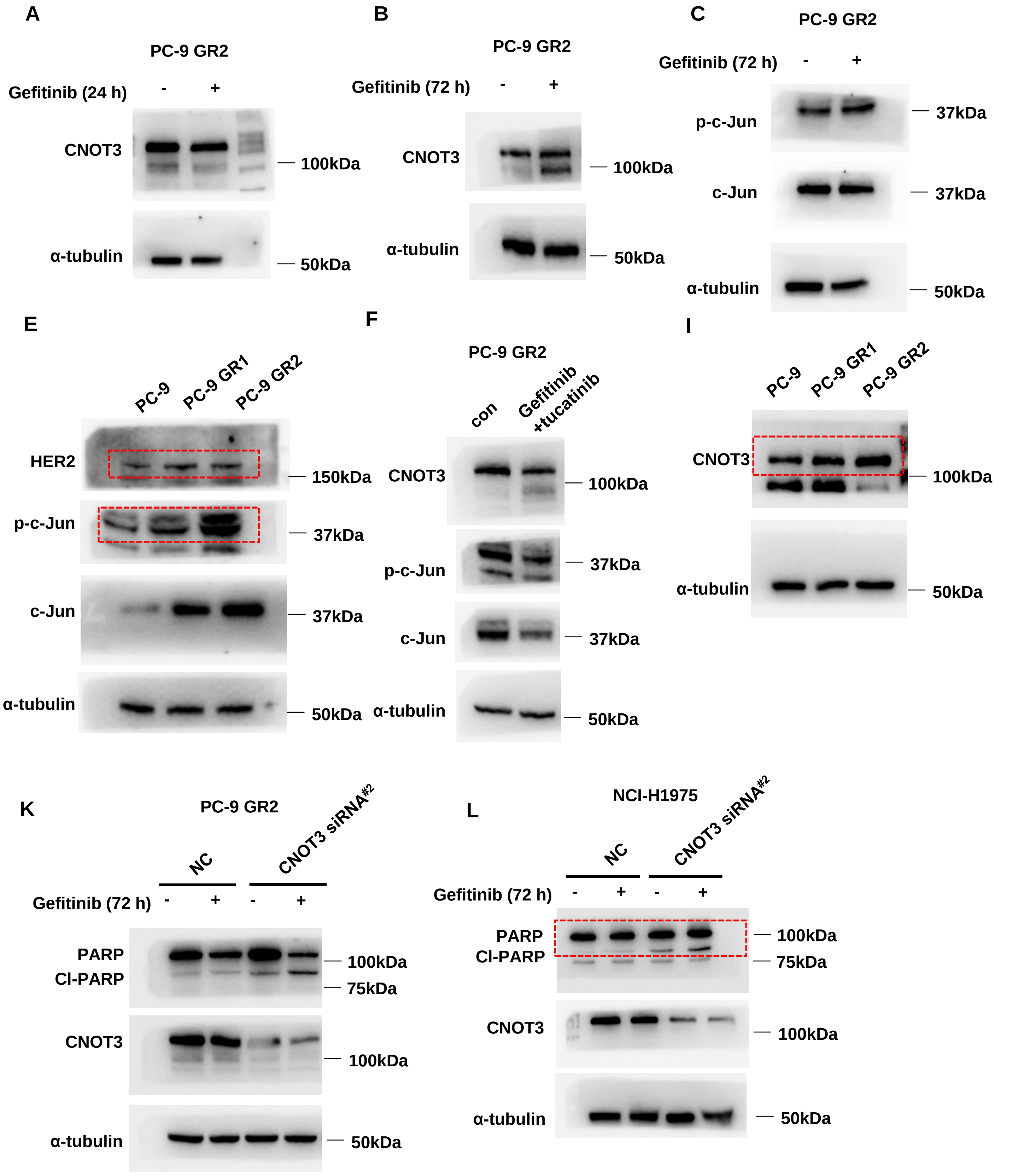
A
PC-9 GR2
Gefitinib (24 h) - +
CNOT3
100kDa
α-tubulin
50kDa
B
PC-9 GR2
Gefitinib (72 h) - +
CNOT3
100kDa
α-tubulin
50kDa
C
PC-9 GR2
Gefitinib (72 h) - +
p-c-Jun
37kDa
c-Jun
37kDa
α-tubulin
50kDa
E
PC-9
PC-9 GR1
PC-9 GR2
HER2
150kDa
p-c-Jun
37kDa
c-Jun
37kDa
α-tubulin
50kDa
F
PC-9 GR2
con
Gefitinib +tucatinib
CNOT3
100kDa
p-c-Jun
37kDa
c-Jun
37kDa
α-tubulin
50kDa
I
PC-9
PC-9 GR1
PC-9 GR2
CNOT3
100kDa
α-tubulin
50kDa
K
PC-9 GR2
NC
CNOT3 siRNA#2
Gefitinib (72 h) - + - +
PARP
Cl-PARP
100kDa
75kDa
CNOT3
100kDa
α-tubulin
50kDa
L
NCI-H1975
NC
CNOT3 siRNA#2
Gefitinib (72 h) - + - +
PARP
Cl-PARP
100kDa
75kDa
CNOT3
100kDa
α-tubulin
50kDa

Supplementary Figure 2

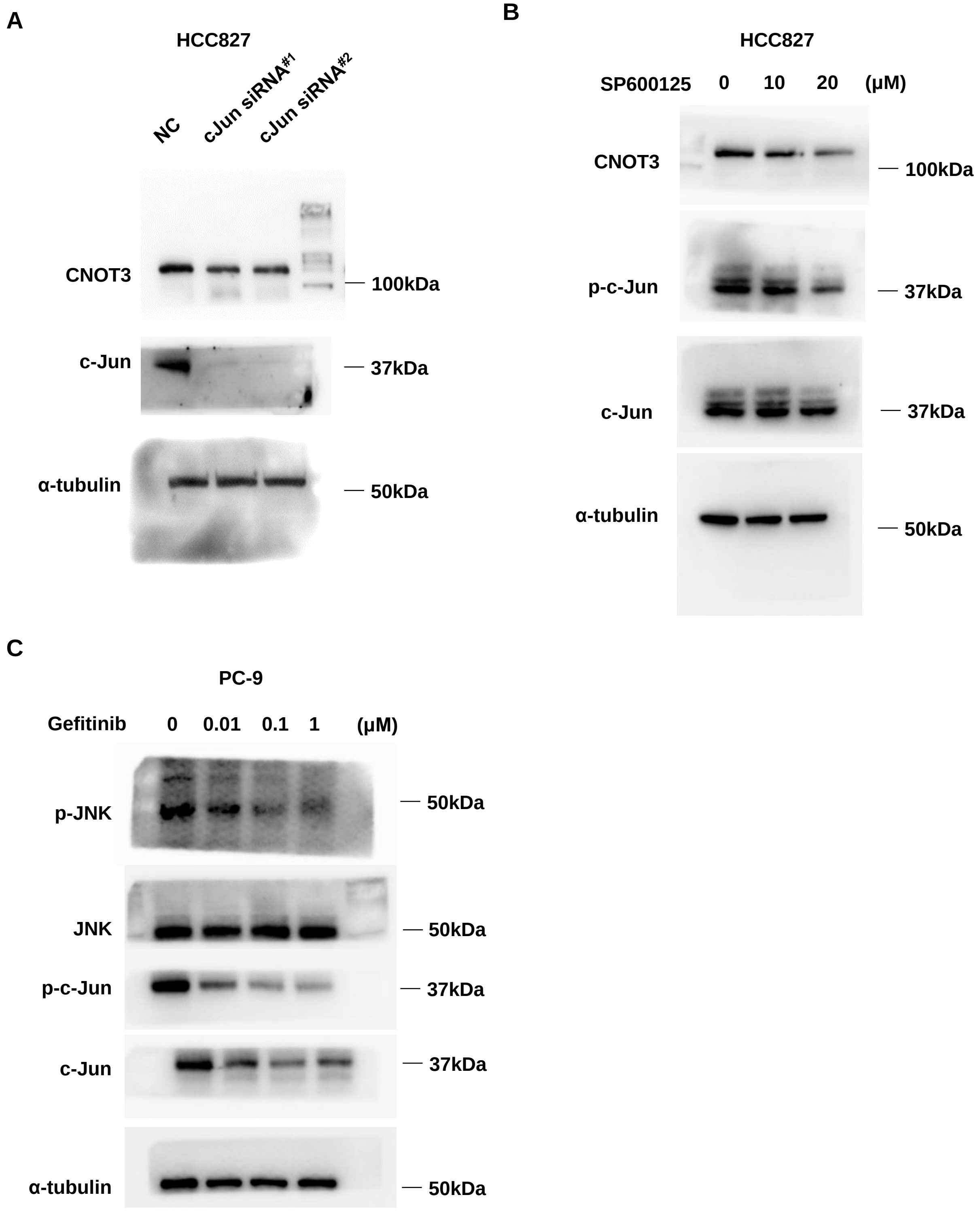

Supplementary Figure 3

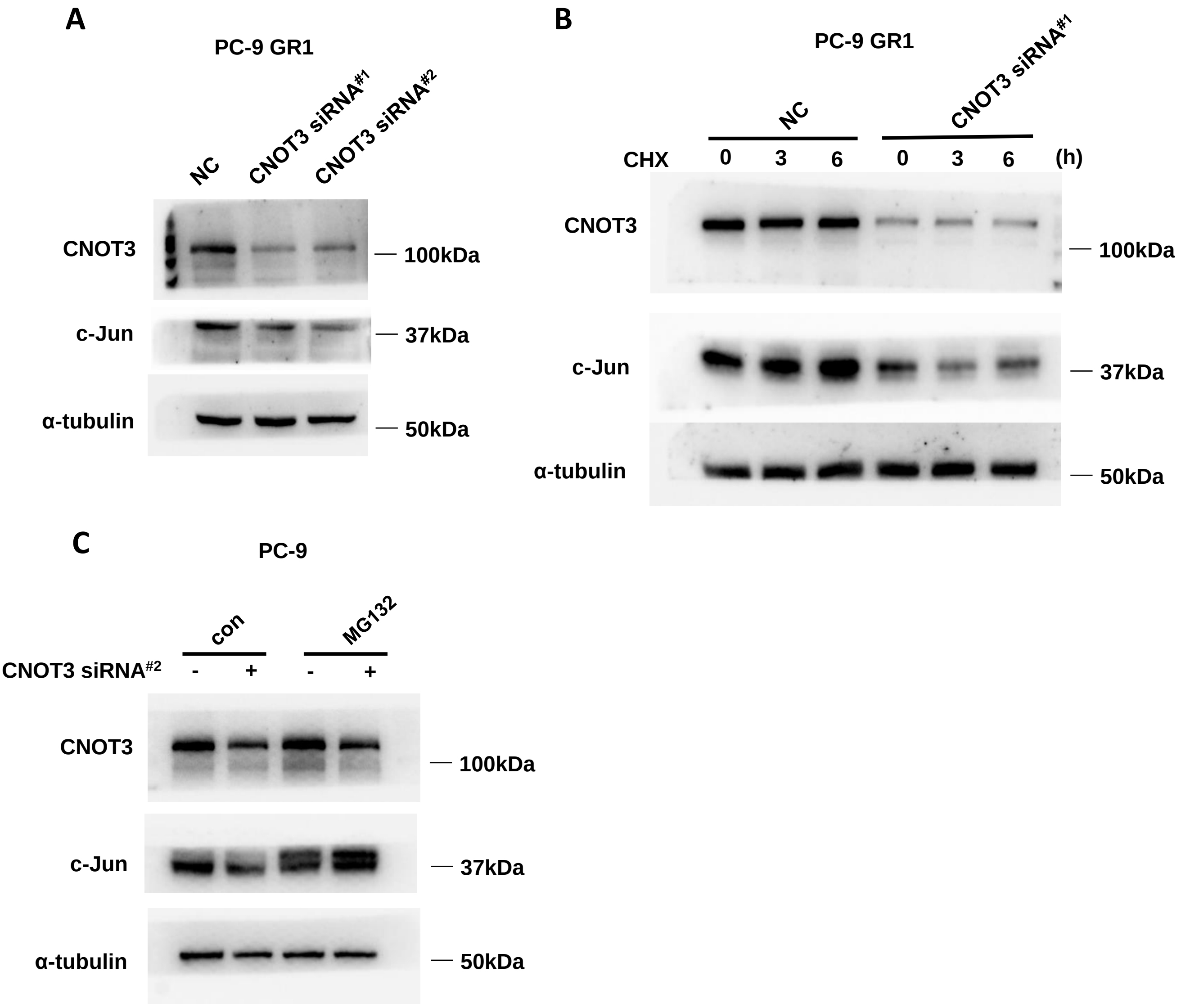

Supplementary Figure 4

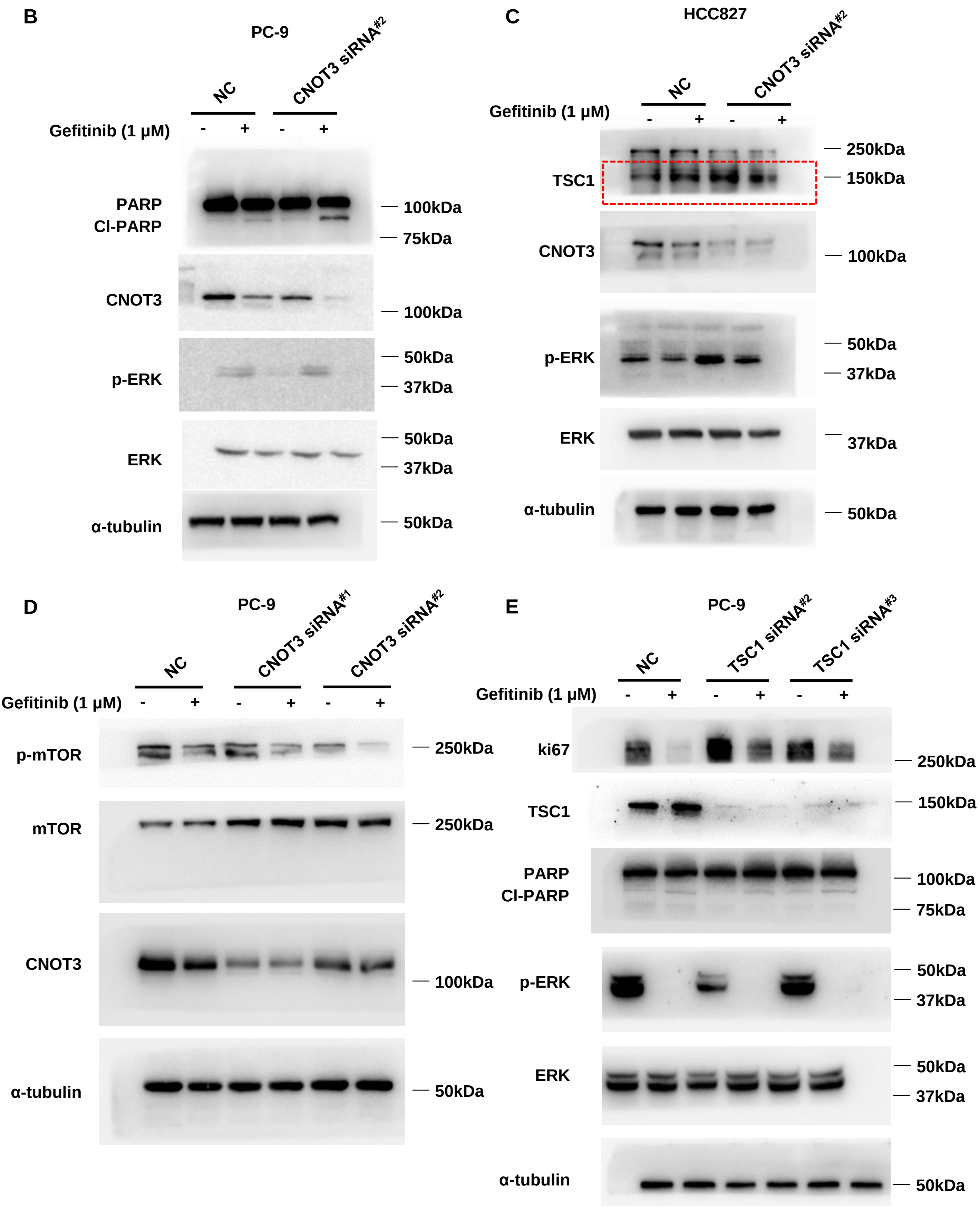

Supplementary Figure 5

C

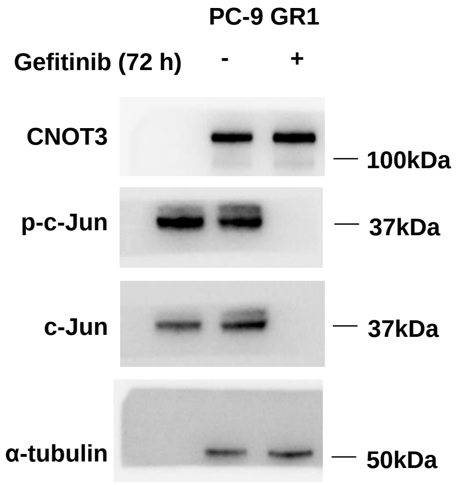

E

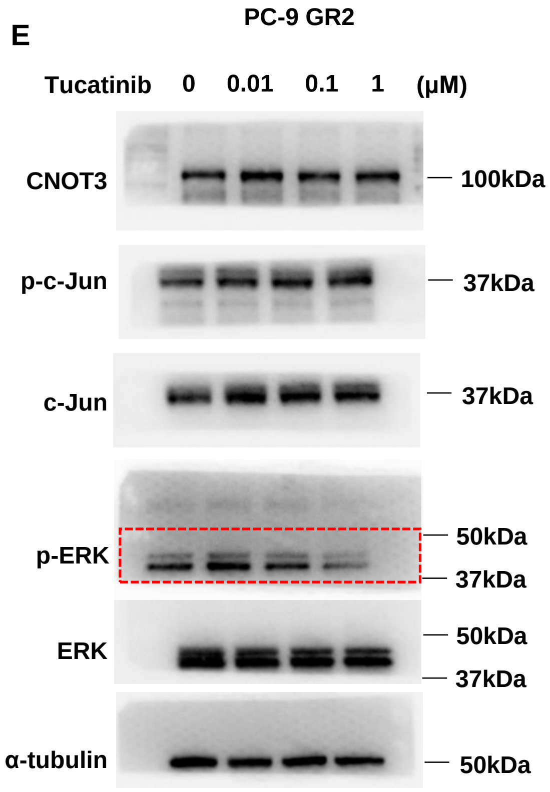

Figure 6

A

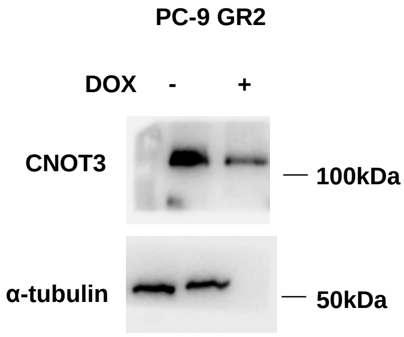

Supplementary Figure 6

B

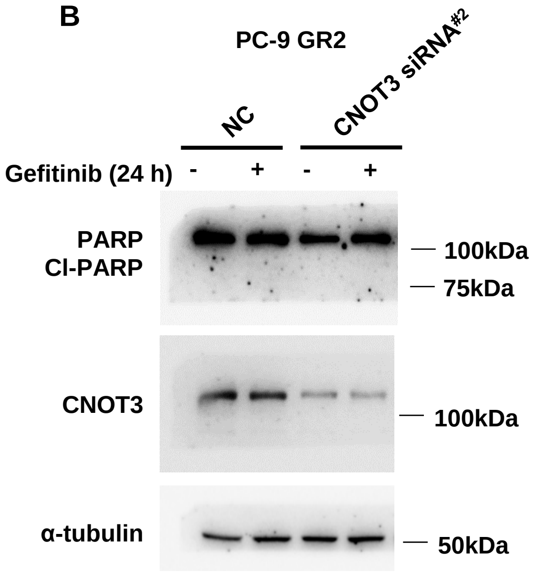

F

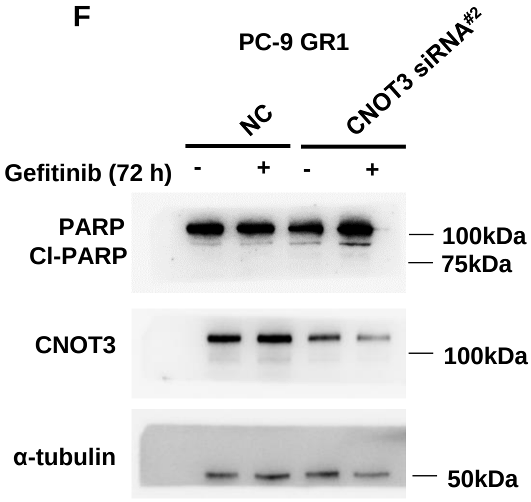

Supplement: Supplementary file 9 — western blotting uncropped [file 41420_2023_1701_MOESM9_ESM.pdf]
